# GenToS: Use of Orthologous Gene Information to Prioritize Signals from Human GWAS

**DOI:** 10.1371/journal.pone.0162466

**Published:** 2016-09-09

**Authors:** Anselm S. Hoppmann, Pascal Schlosser, Rolf Backofen, Ekkehart Lausch, Anna Köttgen

**Affiliations:** 1 Dept. of Pediatric Genetics, Medical Center – University of Freiburg, Faculty of Medicine, University of Freiburg, Freiburg, Germany; 2 Division of Genetic Epidemiology, Institute for Medical Biometry and Statistics, Medical Center – University of Freiburg, Faculty of Medicine, University of Freiburg, Freiburg, Germany; 3 Institute for Medical Biometry and Statistics, Medical Center – University of Freiburg, Faculty of Medicine, University of Freiburg, Freiburg, Germany; 4 Bioinformatics Group, Department of Computer Science, University of Freiburg, Freiburg, Germany; McMaster University, CANADA

## Abstract

Genome-wide association studies (GWAS) evaluate associations between genetic variants and a trait or disease of interest free of prior biological hypotheses. GWAS require stringent correction for multiple testing, with genome-wide significance typically defined as association p-value <5*10^−8^. This study presents a new tool that uses external information about genes to prioritize SNP associations (GenToS). For a given list of candidate genes, GenToS calculates an appropriate statistical significance threshold and then searches for trait-associated variants in summary statistics from human GWAS. It thereby allows for identifying trait-associated genetic variants that do not meet genome-wide significance. The program additionally tests for enrichment of significant candidate gene associations in the human GWAS data compared to the number expected by chance. As proof of principle, this report used external information from a comprehensive resource of genetically manipulated and systematically phenotyped mice. Based on selected murine phenotypes for which human GWAS data for corresponding traits were publicly available, several candidate gene input lists were derived. Using GenToS for the investigation of candidate genes underlying murine skeletal phenotypes in data from a large human discovery GWAS meta-analysis of bone mineral density resulted in the identification of significantly associated variants in 29 genes. Index variants in 28 of these loci were subsequently replicated in an independent GWAS replication step, highlighting that they are true positive associations. One signal, *COL11A1*, has not been discovered through GWAS so far and represents a novel human candidate gene for altered bone mineral density. The number of observed genes that contained significant SNP associations in human GWAS based on murine candidate gene input lists was much greater than the number expected by chance across several complex human traits (enrichment p-value as low as 10^−10^). GenToS can be used with any candidate gene list, any GWAS summary file, runs on a desktop computer and is freely available.

## Introduction

Genome-wide association studies (GWAS) are an unbiased approach to identify genomic risk loci for complex diseases and to gain insight into underlying pathogenic mechanisms. Over the past decade, GWAS have led to the identification of previously unknown risk loci for hundreds of traits and diseases [1,2]. To reduce the type I error and account for association testing of an estimated one million common independent single nucleotide polymorphisms (SNPs) in the human genome [3], a multiple testing corrected significance level (alpha of 5*10^−8^ [0.05/1,000,000]) has been adopted in the GWAS community. This rather conservative Bonferroni correction results in an increased type II error: increasingly larger GWAS meta-analyses of the same phenotype have demonstrated that results for a given GWAS meta-analysis contain multiple true positive findings that do not achieve genome-wide significant association p-values. Such associations can then only be identified and replicated at genome-wide significance once sample size is increased in subsequent analyses. However, increasing sample size may not always be feasible due to high costs or because of limited phenotype availability for specific diseases or special populations [4]. Therefore, approaches to identify additional candidate genes among these suggestive but not genome-wide significantly associated loci are needed.

Another challenge in the interpretation of associated loci identified through GWAS is that these loci typically contain several or many genes that each contain associated genetic variants in high linkage disequilibrium, complicating the identification of the causal gene(s) and variant(s) within such loci [5]. Again, additional sources of evidence to aid in the prioritization of association signals would be desirable. Several existing approaches leverage external information for the prioritization of potentially causal genes from GWAS data [6–12]. Many of these previous approaches evaluate enrichment of associated SNPs in gene sets based on pre-defined pathways [13], gene ontology terms [14], tissue expression analysis or functionally similar genes. They integrate information across different cell types and organisms and from sources as heterogeneous as *in vitro* protein-protein and chemical interactions. Another external source of information is animal models of phenotypes analogous to the human phenotype of interest, because of the conservation of gene function across species. The mouse represents a suitable model organism because of the relatively short evolutionary distance between humans and mice and because of a comprehensive and systematic effort to generate knock-out animals and/or cells for all murine genes [15,16]. Previous approaches that have integrated evidence from GWAS and mouse models have focused on evidence from naturally occurring genetic markers for subsequent use in linkage analysis [17] or genome-wide association testing [18].

We aimed to develop a method that provides complementary information to previous approaches by using a comprehensive resource of genetically manipulated and then systematically phenotyped mice (reverse genetics approach) in order to generate biological candidate gene lists. These genes are then evaluated using summary association statistics from GWAS of a corresponding human disease or phenotype. We validate the method across several human complex traits and diseases including bone mineral density, diabetes, glycemic traits and blood pressure phenotypes, and show that genes causing a specific phenotype in mouse models are significantly enriched for associated SNPs in results from GWAS of a corresponding human phenotype. Finally, we show that the method can identify novel candidate genes not claimed by GWAS so far for future validation.

## Results

**Fig 1:**
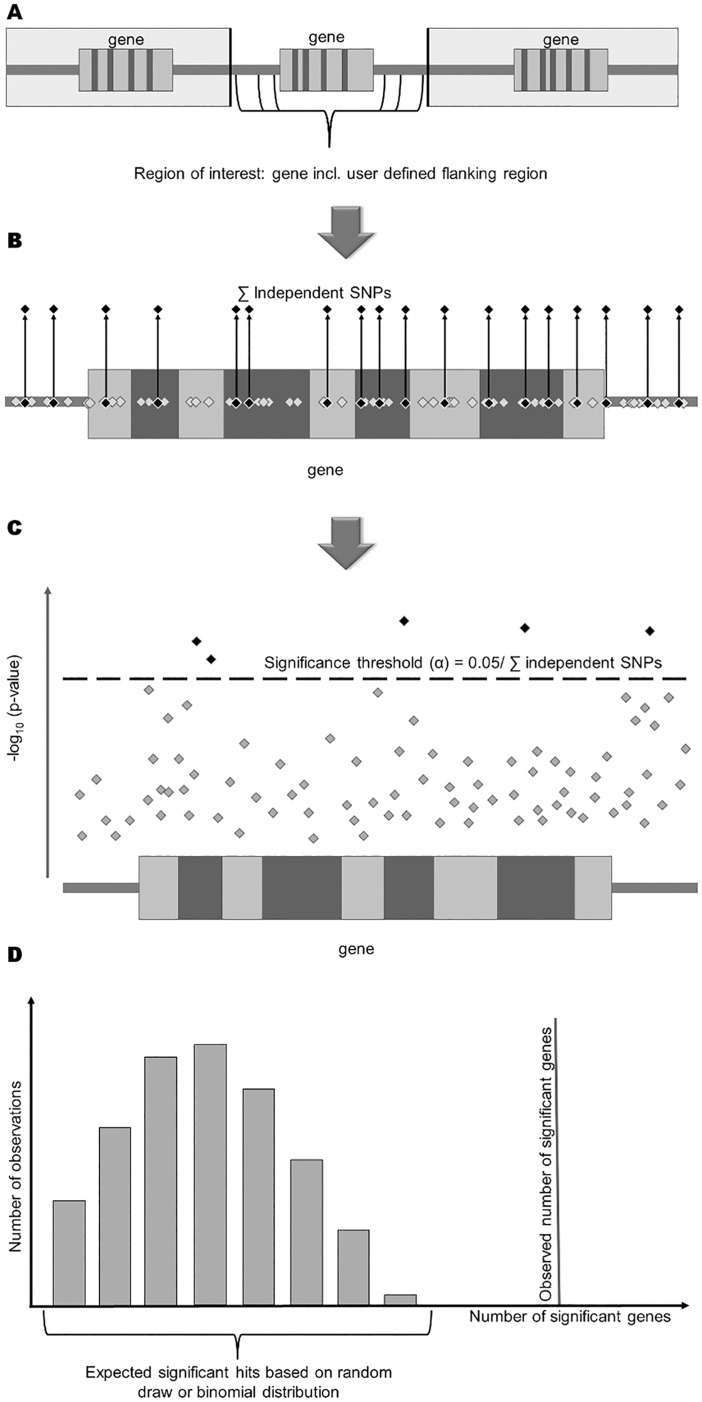
GenToS principle. **(A)** First, GenToS extracts for each gene on a given candidate gene input list the region of the gene including a user-defined flanking region. **(B)** Next, all independent SNPs within each region are identified from a reference population, and a significance threshold based on the number of independent SNPs is calculated. **(C)** In the final step, SNPs with an association p-value below the calculated significance threshold are extracted from the human GWAS summary results. **(D)** Enrichment of the number of observed significant genes (vertical line) can be assessed visually compared to the expected number based on a null distribution derived by resampling from a binomial distribution (histogram).

The GenToS algorithm is built as a three-step procedure. It requires a candidate gene input list that contains gene identifiers of human orthologs of genes causing a specific phenotype in genetically manipulated mice. In a first step, the corresponding genomic coordinates for each gene on the candidate gene input list are obtained (Fig 1A). Next, the number of independent common single nucleotide polymorphisms (SNPs) within each candidate gene region is determined based on a reference population, to subsequently calculate a statistical significance threshold based on the number of independent SNPs across all genes on a list (Fig 1B). Third, all derived gene regions are queried for the presence of SNPs with association p-values below the derived significance threshold in results from a human GWAS of the same or similar phenotype (Fig 1C). In addition to this three-step procedure, a validation step can be performed to examine whether the use of the candidate gene input list leads to the identification of more genes that contain significant associations than expected by chance (enrichment, Fig 1D). Detailed information is provided in the Methods section.

### Enrichment of the number of genes with significant association signals based on a candidate gene input list

Enrichment of significant GWAS associations based on a candidate gene input list can be assessed compared to the null distribution of significant GWAS associations expected by chance. The null distribution can be derived by a resampling approach where each randomly drawn gene input list contains an equal number of genes as the candidate gene input list. Since this iterative procedure is time consuming, we assessed the properties of this distribution. The test of identifying SNPs below the significance threshold for a given gene can be considered a Bernoulli trial. Thus, the number of genes that contain significant GWAS association signals from an input gene list should follow a binomial distribution.

First, 2,000 iterations of GenToS were carried out for each of several fixed statistical significance thresholds (range 1*10^−2^ to 1*10^−8^). For every threshold, each of the 2,000 iterations used an input gene list that contained 1,292 randomly drawn genes, corresponding to the number of genes on the candidate gene input list for abnormal murine skeleton morphology (see next section). The human GWAS summary statistics dataset used to identify significantly associated SNPs was obtained from a meta-analysis of GWAS for bone mineral density (for details, see Methods). For each of the 2,000 iterations, the number of genes from each input list was counted that contained SNPs associated with bone mineral density below the respective significance threshold.

**Fig 2:**
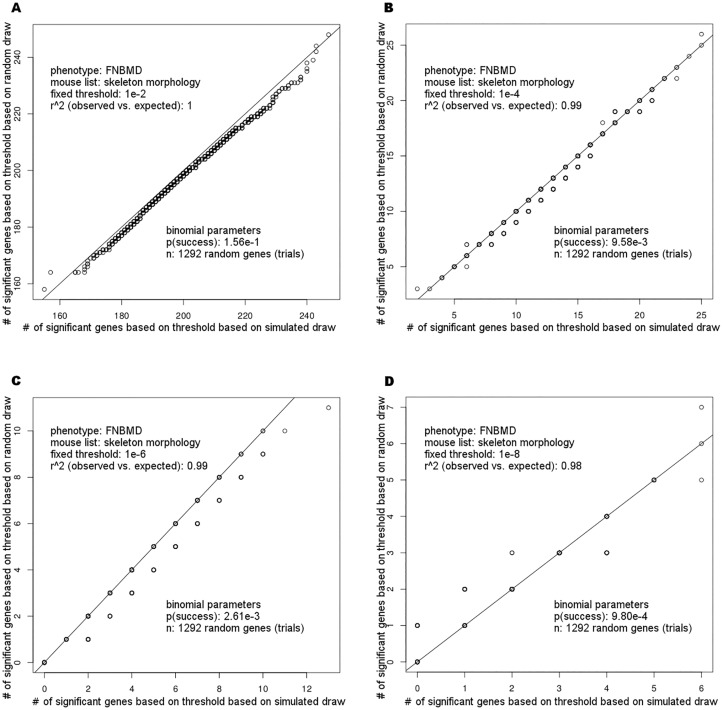
QQ-plots of the number of observed significant genes under the null hypothesis comparing random draws of gene input lists and simulated draws. The graph shows that simulated draws based on a binomial experiment approximate the number of significant genes under the null hypothesis derived from iterations of randomly generated input gene lists, while being computationally more efficient. QQ plots were generated across a range of possible significance thresholds. Spearman correlation coefficients were determined for each setting and found to be in the range of 0.90–1.00.

Next, 2,000 iterations of a binomial experiment were carried out to simulate a binomial distribution. In each of these, p was the probability of observing a significant gene association, estimated by the proportion of genes that contained significant SNP associations below the evaluated fixed significance threshold among all 25,230 entries in the human gene database, and the number of Bernoulli trials n was 1,292, the number of genes in the candidate gene list. After 2,000 iterations of the simulated random draw, the number of significant genes was plotted against the number obtained from the iterative random draw using quantile-quantile (QQ)-plots. Fig 2 shows good agreement of the number of significant genes detected by the two approaches across a range of selected significance thresholds. The QQ plots for all evaluated significance thresholds are shown in S1 Fig for input gene lists that contain as many genes as the abnormal skeleton morphology candidate gene list (the longest candidate gene list) and in S2 Fig for input gene lists that contained 134 genes as the abnormal bone mineralization list (the shortest candidate gene list). Spearman rank-correlation coefficients between the number of significant genes for the two approaches ranged from 0.90–1.00 across all QQ plots. We therefore decided to subsequently use the binomial distribution to visually assess and quantify enrichment of human GWAS association signals based on candidate gene input lists. Enrichment p-values were estimated using a complementary cumulative binomial distribution (see Methods).

### GWAS of human skeletal phenotypes are enriched for signals in genes causing bone phenotypes in mouse models

**Fig 3:**
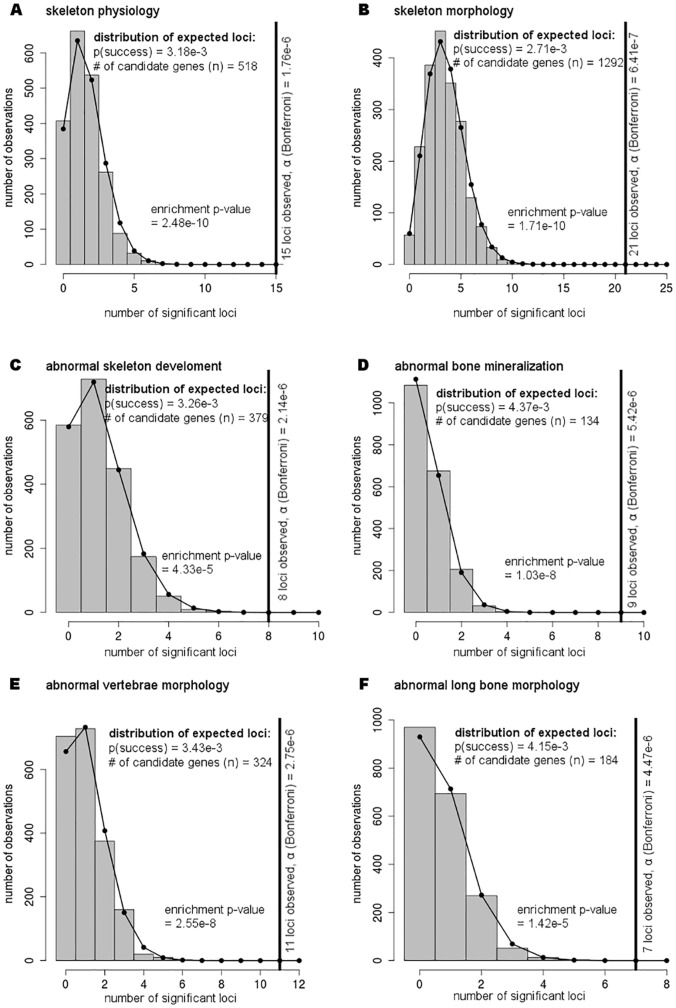
GenToS identifies significant enrichment of genes containing femoral neck bone mineral density-associated SNPs based on candidate gene input lists for murine bone phenotypes. For each of the six candidate gene input lists, the number of expected significant genes under the null hypothesis was generated based on iterations of randomly drawn gene lists that contained an equal number of genes as the respective candidate gene input list and is displayed as a histogram. In addition, the binomial density distribution corresponding to the candidate gene input list significance threshold was overlaid (dots connected with lines). The observed number of significant genes based on the use of GenTos with the candidate gene input lists and the human GWAS results for femoral neck bone mineral density is indicated by a vertical black line. The enrichment p-value is computed from the complementary cumulative binomial distribution (see Methods).

Using publicly available summary statistics from the discovery stage of GWAS meta-analyses for femoral neck bone mineral density (FNBMD) and lumbar spine bone mineral density (LSBMD) of the GEFOS Consortium [19,20], GenToS was used to test for enrichment of GWAS association signals in genes that give rise to six different skeletal phenotypes in mouse models. Depending on which of the six candidate gene input lists was used (see Methods), a range of 6–21 significant genes were identified in human GWAS based on the Bonferroni method to derive the significance threshold (see Methods). The number of significant genes was higher than that expected by chance for each candidate gene input list, with enrichment p-values ranging from 2.62*10^−3^ to 1.71*10^−10^ depending on the human phenotype (FNBMD or LSBMD) and the mouse candidate gene input list. Fig 3 shows the observed number of genes that contained significant associations compared to 2,000 randomly drawn input gene lists that contained an equal number of genes as the candidate gene input list, as well as the enrichment p-values for each of the six evaluated candidate gene input lists in relation to FNBMD. Results were also significant and very similar for LSBMD (S3 Fig).

Across all six candidate gene input lists and the two human phenotypes, 29 unique genes contained significantly associated SNPs (Tables 1 and 2). The greatest number of genes, 21, was found in association with FNBMD using the longest and rather general candidate gene input list, “abnormal skeleton morphology” (enrichment p-value of 1.71*10^−10^, Fig 3).

**Table 1:** Genes identified by GenToS in association with human bone mineral density phenotypes that reached genome-wide significance and were replicated in previous GWAS.

Gene	Cyto-band	index SNP, FNBMD	p-value, FNBMD	index SNP, LSBMD	p-value, LSBMD	in GWAS Catalog*	OMIM number	monogenic phenotype
***WLS***	1p31.3	rs1430742	2.91E-13	rs878548	1.52E-19	Estrada K, rs12407028-T, 3 x 10–45 (LSBMD)		
***SPTBN1***	2p16.2			rs11898505	9.72E-12	Estrada K, rs4233949-C, 2 x 10–18 (LSBMD)		
***PKDCC***	2q21	rs13005448	7.99E-07			Estrada K, rs7584262-T, 1 x 10–9 (FNBMD)		
***GALNT3***	2q24.3	rs1346004	1.62E-10	rs1346004	4.44E-08	Estrada K, rs1346004-A, 4 x 10–30 (LSBMD); Duncan EL, rs6710518-T, 5 x 10–10 (femoral neck)	211900	Tumoral calcinosis, hyperphosphatemic, familial
***IDUA***	4p16.3	rs3755955	3.73E-07			Estrada K, rs3755955-A, 5 x 10–15 (LSBMD)	607014; 607015; 607016	Mucopolysaccharidosis Ih; Mucopolysaccharidosis Ih/s; Mucopolysaccharidosis Is
***MEPE***	4q22.1	rs1054629	9.23E-10	rs1471403	1.67E-10	Estrada K, rs6532023-T, 1 x 10–27 (LSBMD); Zhang L, rs1463104-?, 2 x 10–9 (spine)		
***MEF2C***	5q14.3	rs17558396	6.54E-07			Estrada K, rs1366594-A, 4 x 10–61 (FNBMD); Zhang L, rs6894139-?, 7 x 10–18 (FNK); Zheng HF, rs11951031-T, 9 x 10–9; Duncan EL, rs6710518-T, 8 x 10–10 (femoral neck)	613443; 613443	Chromosome 5q14.3 deletion syndrome; Mental retardation, stereotypic movements, epilepsy, and/or cerebral malformations
***ESR1***	6q25.1	rs3020331	1.30E-14	rs2941741	1.19E-14	Estrada K, rs4869742-T, 4 x 10–35 (LSBMD); Paternoster L, rs6909279-G, 1 x 10–9 (Cortical vBMD)	615363; 114480; 157300; 608446	Estrogen resistance; {Breast cancer}; {Migraine, susceptibility to}; {Myocardial infarction, susceptibility to}; {Breast cancer} (no OMIM); {Migraine, susceptibility to} (no OMIM)
***WNT16***	7q31.31	rs3801387	4.67E-14	rs3801387	1.58E-15	Estrada K, rs3801387-A, 3 x 10–51 (LSBMD); Zhang L, rs10242100-?, 2 x 10–10 (hip)		
***TNFRSF11B***	8q24.12	rs4242592	1.58E-14	rs10505348	3.22E-18	Estrada K, rs2062377-A, 3 x 10–39 (LSBMD); Zhang L, rs4424296-?, 9 x 10–14 (spine); Richards JB, rs4355801-A, 8 x 10–10; Paternoster L, rs2062377-A, 1 x 10–7 (Cortical vBMD)	239000	Paget disease of bone 5, juvenile-onset
***ARHGAP1***	11p11.2	rs7932354	2.62E-08			Estrada K, rs7932354-T, 5 x 10–18 (FNBMD)		
***SOX6***	11p15.2-p15.1	rs4757390	3.94E-07			Estrada K, rs7108738-T, 1 x 10–32 (FNBMD); Zhang L, rs7108738-?, 1 x 10–15 (FNK)		
***LRP5***	11q13.2	rs608343	5.77E-07	rs3736228	1.32E-10	Estrada K, rs3736228-T, 2 x 10–26 (LSBMD); Richards JB, rs3736228-T, 6 x 10–12; Zhang L, rs525592-?, 3 x 10–11 (spine)	601813; 144750; 607634; 259770; 144750; 607636; 601884; 166710	Exudative vitreoretinopathy 4; Hyperostosis, endosteal; Osteopetrosis, autosomal dominant 1; Osteoporosis-pseudoglioma syndrome; Osteosclerosis; van Buchem disease, type 2; [Bone mineral density variability 1]; {Osteoporosis}
***HOXC6***	12q13.13	rs10876528	1.23E-07	rs894737	6.38E-10	Estrada K, rs736825-C, 8 x 10–16 (LSBMD)		
***SP7***	12q13.13	rs2016266	5.44E-07	rs2016266	3.97E-12	Estrada K, rs2016266-A, 3 x 10–20 (LSBMD)	613849	?Osteogenesis imperfecta, type XII
***AXIN1***	16p13.3	rs9921222	2.22E-07	rs9921222	7.26E-08	Estrada K, rs9921222-T, 1 x 10–16 (LSBMD)	607864; 114550	?Caudal duplication anomaly; Hepatocellular carcinoma, somatic
***CLCN7***	16p13.3	rs13336428	2.55E-07			Estrada K, rs13336428-A, 1 x 10–16 (FNBMD)	166600; 611490	Osteopetrosis, autosomal dominant 2; Osteopetrosis, autosomal recessive 4
***SOST***	17q21.31	rs2741856	5.10E-08	rs2741856	1.29E-07	Estrada K, rs4792909-T, 2 x 10–11 (FNBMD)	122860; 269500; 239100	Craniodiaphyseal dysplasia, autosomal dominant; Sclerosteosis 1; Van Buchem disease
***TNFRSF11A***	18q21.33			rs884205	2.62E-08	Estrada K, rs884205-A, 2 x 10–17 (LSBMD)	174810; 612301; 602080	Osteolysis, familial expansile; Osteopetrosis, autosomal recessive 7; {Paget disease of bone 2, early-onset}
***JAG1***	20p12.2	rs6514116	8.88E-08	rs6040061	9.23E-10	Estrada K, rs3790160-T, 3 x 10–19 (LSBMD); Kung AW, rs2273061-A, 5 x 10–8	118450; 187500	Alagille syndrome; Tetralogy of Fallot; Deafness, congenital heart defects, and posterior embryotoxon (non OMIM)

The index SNP is defined as the SNP with the lowest association p-value with a given trait. The GWAS Catalog entry refers to results obtained from the NHGRI GWAS catalog upon entry of the given index SNP. Monogenic phenotypes are retrieved from OMIM. Of note, several of these genes only achieved genome-wide significance after the replication step, whereas GenToS is based on data from the discovery step and already implicated the genes at this point. Empty cells for LSBMD and MNBMD p-values and SNP identifiers indicate that no SNP in the gene contained significant associations below any of the six murine candidate gene list-wise thresholds.

*LSBMD and FNBMD entries from the GWAS catalog represent summary estimates from the combined discovery and replication step.

LSBMD = Lumber spine bone mineral density; FNBMD = Femoral neck bone mineral density; OMIM = Online Mendelian Inheritance in Man database

**Table 2:** Newly implicated genes identified by GenToS in association with bone mineral density phenotypes. These genes either mapped into known associated GWAS regions but were not previously named as the index gene, or were not replicated at genome-wide significance at the time the GWAS data was published.

Gene	Cyto-band	index SNP, FNBMD	p-value, FNBMD	index SNP, LSBMD	p-value, LSBMD	in GWAS Catalog*	Info	OMIM number	mongenic phenotype	Additional annotation information based on SniPA
***COL11A1***	1p21.1	rs11809524	1.41E-06			Estrada K, rs11809524-T, 9 x 10–6 (FNBMD)		228520; 154780; 604841; 603932	Fibrochondrogenesis 1; Marshall syndrome; Stickler syndrome, type II; {Lumbar disc herniation, susceptibility to}	
***IBSP***	4q22.1	rs1054629	9.228E-10	rs17711209	1.42E-06	Duncan EL, rs1054627-G, 8 x 10–7 (femoral neck)	proximity to *MEPE* (Estrada et al.)			*IBSP* missense variant (transcript 1, FNBMD); Cis-eQTL for *IBSP* and other genes, various tissues (transcript 2, FNMBD); Cis-eQTL for other genes (LSBMD)
***LRP4***	11p11.2	rs6485702	2.605E-07				proximity to *ARHGAP1* (Estrada et al.)	616304; 212780; 614305	?Myasthenic syndrome, congenital, 17; Cenani-Lenz syndactyly syndrome Sclerosteosis 2	*LRP4* missense variant (transcript 1); Cis-eQTL for *LRP4* and other genes, various tissues (transcript 2)
***F2***	11p11.2	rs2070852	3.164E-08				proximity to *ARHGAP1* (Estrada et al.)	613679; 613679; 188050; 614390; 601367	Dysprothrombinemia; Hypoprothrombinemia; Thrombophilia due to thrombin defect; {Pregnancy loss, recurrent, susceptibility to, 2}; {Stroke, ischemic, susceptibility to}	Cis-eQTL for *LRP4*, *ARHGAP1*, and other genes; Splice site region of *F2* in various transcripts; Noncoding exon variant in one transcript
***FGFRL1***	4p16.3	rs6827815	3.966E-06	rs6827815	3.13E-06	Zhang L, rs6827815, 5 x 10–12				upstream gene variant; Putative effect on regulation; Cis-eQTL for different genes
***HOXC10***	12q13.13	rs11614913	6.981E-08	rs11614913	8.92E-10		proximity to *HOXC6* (Estrada et al.)			Cis-eQTL for *HOXC6*, *HOXC8* and other genes; Putative effect on regulation for *HOXC10*, *HOXC6*, *HOXC9* and other genes
***HOXC4***	12q13.13	rs10876528	1.226E-07	rs894737	6.38E-10		proximity to *HOXC6* (Estrada et al.)			Cis-eQTL for *HOXC6*, *HOXC8* and other genes (FNBMD); Putative effect on regulation for *HOXC4*, *HOXC6* and other genes (FNBMD); Cis-eQTL for *HOXC6*, *HOXC8* and other genes (LSBMD); Putative effect on transcript *HOXC4*, *HOXC6* and other genes (LSBMD)
***HOXC8***	12q13.13			rs12300425	1.23E-06		proximity to *HOXC6* (Estrada et al.)			Cis-eQTL for *HOXC9* and other genes; Putative effect on transcript for *HOXC6*, *HOXC9* and other genes
***HOXC9***	12q13.13	rs11614913	6.981E-08	rs11614913	8.92E-10		proximity to *HOXC6* (Estrada et al.)			Cis-eQTL for *HOXC6*, *HOXC8* and other genes; Putative effect on regulation for *HOXC10*, *HOXC6*, *HOXC9* and other genes

The index SNP is defined as the SNP with the lowest association p-value with a given trait. The GWAS Catalog entry refers to results obtained from the NHGRI GWAS catalog upon entry of the given index SNP. Monogenic phenotypes are retrieved from OMIM. Empty cells for LSBMD and MNBMD p-values and SNP identifiers indicate that no SNP in the gene contained significant associations below any of the six murine candidate gene list-wise thresholds.

*LSBMD and FNBMD entries from the GWAS catalog represent summary estimates from the combined discovery and replication step.

SNiPA was used to retrieve cis-eQTL evidence from numerous tissues. Evidence is indicated when any tissue showed indication of an eQTL.

LSBMD = Lumber spine bone mineral density; FNBMD = Femoral neck bone mineral density; OMIM = Online Mendelian Inheritance in Man database

### GenToS identifies novel gene associations for human skeletal phenotypes

Of the 29 genes that contained SNPs significantly associated with human skeletal phenotypes, 20 were published as genome-wide significant loci by the GEFOS Consortium (Table 1) [19,20]. Of these, only 12 had reached genome-wide significance during the GWAS discovery stage, which is used for GenToS, whereas eight additional genes only achieved genome-wide significance after the replication stage of the study. Further, seven of the 29 genes mapped into significant and subsequently replicated GEFOS loci, but had not been named as the gene underlying the association signal in a given locus (Table 2 and S4 Fig). The remaining genes identified by GenToS had not reached genome-wide significance after discovery and replication at the time of the GEFOS publication. One of them, *FGFRL1*, was later identified in a bone mineral density study by Zhang *et al* [21]. The last gene, *COL11A1*, has not been identified by bone-related GWAS to date and thus represents a novel human candidate gene for altered bone mineral density. Altogether, index SNPs in 28 of 29—or >95% of significant genes identified using GenToS with the GEFOS discovery stage data—were subsequently replicated, supporting them as true association signals. Among the genes not previously identified through GWAS or not implicated as the index gene in an associated locus, *LRP4* and *COL11A1* are known to harbor rare mutations that cause monogenic skeletal disease in humans (Table 2). Thus, additional evidence like Cenani-Lenz syndactylyl syndrome or fibrochondrogenesis-1 and the association between the index SNP in *LRP4* and *LRP4* transcript abundance strongly support that the genes identified using GenToS may be the causal one or represent an additional phenotype-associated gene in an associated locus (Table 1).

### Significant associations with additional human phenotypes

To assess whether the enrichment of GWAS signals for genes causing corresponding or related phenotypes in mouse models can be generalized to phenotypes other than human bone mineral density, we explored additional human traits for which GWAS summary statistics are publicly available. This evaluation showed the GenToS approach to be generalizable (Table 3), but that the observed enrichment varied depending on the human phenotype and the input candidate gene list.

**Table 3:** Ontology terms and number of genes in each murine input gene list.

**skeletal phenotype gene lists**			
**name of mouse list**	**ontology term**	**# of genes in list**	**# of genes after filterting**
abnormal skeleton physiology	MP:0005508	518	498
abnormal skeleton morphology	MP:0001533	1292	1247
abnormal skeleton development	MP:0002113	379	366
abnormal bone mineralization	MP:0002113	134	128
abnormal vertebrae morphology	MP:0000137	324	317
abnormal long bone morphology	MP:0003723	184	180
**glucose gene lists**			
**name of mouse list**	**ontology term**	**# of genes in list**	
abnormal circulating glucose level	MP:0000188	560	543
abnormal fasted circulating glucose level	MP:0013277	60	60
decreased circulating glucose level	MP:0005560	324	315
decreased fasted circulating glucose level	MP:0013278	21	21
increased circulating glucose level	MP:0005559	272	263
increased fasted circulating glucose level	MP:0013279	44	44
**insulin gene lists**			
**name of mouse list**	**ontology term**	**# of genes in list**	
abnormal circulating insulin level	MP:0001560	385	373
abnormal insulin secretion	MP:0003564	147	144
increased insulin secretion	MP:0003058	42	39
decreased circulating insulin level	MP:0002727	240	234
decreased insulin secretion	MP:0003059	110	109
increased circulating insulin level	MP:0002079	163	157
**blood pressure gene lists**			
**name of mouse list**	**ontology term**	**# of genes in list**	
increased systemic arterial blood pressure	MP:0002842	131	128
increased systemic arterial systolic blood pressure	MP:0006144	67	65
decreased systemic arterial blood pressure	MP:0002843	92	87
decreased systemic arterial systolic blood pressure	MP:0006264	39	36
**diabetes gene lists**			
**name of mouse list**	**ontology term**	**# of genes in list**	
hyperglycemia	MP:0001559	99	93
abnormal glucose tolerance	MP:0005291	406	394

For each list, <5% of genes were filtered, mostly because they were mapping to human gonosomes and gonosomal GWAS summary statistics were not available. Other reasons for filtering included ambiguous mapping and accounted for <1% of filtered genes for each list.

For type 2 diabetes, studied in 57,000 participants of the DIAGRAM Consortium [22], enrichment of genes that contained significantly associated SNPs was observed for two of the candidate gene input lists (S5 Fig): for the list of candidate genes that when modified cause “hyperglycemia” in mouse models, five significant genes were identified in the DIAGRAM data (enrichment p-value 3.11*10^−5^, S1 Table). For the candidate gene list “abnormal glucose tolerance”, seven significant genes were found (enrichment p-value 6.54*10^−6^, S1 Table).

For systolic blood pressure, human GWAS summary data from the ICBP Consortium was used (n = 74,000 [23,24]), and 4 different candidate gene input lists were tested (see Methods). None of the tested candidate gene lists showed nominally significant enrichment for association signals in humans (S6 Fig, S1 Table), although the number of genes with significant association signals in the lists “increased systemic arterial blood pressure” and “decreased systemic arterial blood pressure” approached statistical significance.

Finally, glycemic traits studied in the MAGIC Consortium were evaluated. For association with the human trait fasting insulin concentrations (GWAS data based on 38,000 individuals [25]), six different candidate gene input lists ranging from 42 to 385 genes were evaluated (see Methods). Nominally significant enrichment of associated genes was identified for two candidate gene lists (S7 Fig), “abnormal circulating insulin level” (enrichment p-value 3.21*10^−2^) and “increased circulating insulin level” (enrichment p-value 2.05*10^−2^, with associated genes listed in S1 Table. All other candidate gene lists did not give rise to any significant association signals in humans. The other human trait evaluated was fasting glucose (GWAS data for 46,000 individuals [25]). Six different candidate gene input lists were evaluated, representing three mouse traits, each in the fasting and non-fasting state. Significant enrichment of the number of genes that contained association signals in humans was only observed for the non-fasting candidate gene input lists (S8 Fig): 6 significant genes were identified for “abnormal circulating glucose level” (enrichment p-value 5.12*10^−4^), 3 for”decreased circulating glucose level” (enrichment p-value 2.49*10^−2^), and 6 for”increased circulating glucose level (enrichment p-value 2.41*10^−5^), with associated genes shown in S1 Table. Conversely, no enrichment and in fact no significant genes at all were identified for the candidate gene input lists from the fasting counterpart of the murine phenotype.

## Discussion

In this study we introduced GenToS, a tool to prioritize genes from GWAS summary statistics using candidate gene information obtained from another species, the mouse. We show across a variety of complex diseases/traits that GenToS identifies significant enrichment of GWAS association signals in the human orthologs of these candidate genes. The potential of the method is illustrated by the fact that—using bone phenotypes as exemplary data—more than 95% of the genes identified by GenToS were replicated as true positives in a replication step or subsequent studies. Our findings underline the high functional conservation of genes between mice and humans and suggest that the incorporation of murine data can be particularly helpful when further increases in sample size for human GWAS cannot easily be achieved.

There are several other tools to prioritize potentially causal genes in associated loci originating from human GWAS [6,11,12,26–28]. An approach taken by programs like DEPICT [11], MAGENTA [28] INRICH [27] and PARIS [12] is to evaluate enrichment of associated SNPs in gene sets based on pathways, tissue expression analysis or functionally similar genes. These gene sets are typically based on pre-existing Gene Ontology terms [14] or KEGG pathways [13], which integrate information across different cell types and organisms and from sources as heterogeneous as *in vitro* protein-protein and chemical interactions. GenToS on the other hand uses gene sets composed of biological candidate genes based on the systematic generation and grouping of observed phenotypes in the mouse, a widely used model organism to study human disease. Thus, pathway-based analyses and the approach implemented in GenToS provide complementary information.

With respect to using mouse models as the primary source of information for the selection of candidate genes, our approach is complementary to a recently published method by Wang *et al*. [18]. The approach by Wang *et al* used naturally occurring genetic variants in recombinant inbred mouse strains for association testing with multiple murine (endo-) phenotypes, followed by examination of selected, implicated genes across many phenotypes in a human population genotyped only for the coding portion of the genome (exome chip). Our approach on the other hand uses genetically manipulated mice that feature a specific phenotype, followed by combination with results from a genome-wide genetic screen of a corresponding phenotype in humans. Our approach is therefore more focused in that it concentrates on specific and analogous rather than hundreds of phenotypes as well as on genetic manipulations of strong effect (e.g., complete gene knockouts), which can facilitate the interpretation of findings. In addition, the focus on one or a few related phenotypes allows for the derivation of a conservative multiple-testing corrected significance threshold in GenToS, which is difficult to establish in a phenome-wide context, as discussed by the authors [18]. Conversely, the approach by Wang and colleagues allows for discovering novel cross-phenotype associations and for assessing the effects of naturally occurring, hypomorphic genetic variants. The latter should theoretically enable the study of regulatory variants, although the authors chose to study only 12,000 high-impact (missense, nonsense, splice, frameshift, CNVs) out of 5 million discovered genetic variants. For many of these high-impact variants, no associated murine phenotype was observed, which can be explained by mechanisms such as compensation or by incomplete phenotype availability. Finally, the use of GWAS in our approach allows for the identification of associated SNPs that map into introns and gene regulatory regions, whereas the approach by Wang *et al* only focused on human genetic variants in the coding portion of the genome (exome chip). Thus, the evidence generated by the two approaches can be considered complementary.

The comparison of GenToS results across different candidate gene input lists and GWAS summary statistics datasets allows for several observations: first, the strength of enrichment did not increase when the murine phenotype was selected as closely as possible to the phenotype for which human GWAS association statistics were available. This is illustrated by the fact that the enrichment for genes on the rather general murine candidate gene list for skeleton morphology was stronger than that for the more specific murine candidate gene list for abnormal bone mineralization, the phenotype studied in humans. Second, findings across related human traits were very similar, as evidenced by the comparison of GWAS of femoral neck and lumbar spine bone mineral density. Third, our observation of significant enrichment was generalizable to non-skeletal phenotypes, as exemplified by significant enrichment for association signals in murine candidate genes for abnormal insulin levels and hyperglycemia in the corresponding human traits.

It is noteworthy that the significance of the observed enrichment varied across the examined phenotypes/diseases. There are several potential explanations for this observation: firstly, the genetic architecture of the examined phenotypes can differ. Whereas susceptibility to one disease may be explained by variants of large effect in relatively few genes, variants of small effect in several hundreds of genes may contribute to other diseases, requiring better-powered i.e. larger GWAS for their detection. Secondly, the publicly available data used in this report varied in sample size, thereby preventing a comparison of phenotypes at a fixed GWAS sample size. Thirdly, the phenotypic characterization in mice is not equally easy or complete across phenotypes. For instance, abnormal bone morphology in knockout mice is more easily observed than phenotypes requiring invasive measurements such as the recording of blood pressure, which may in addition be subject to biological variation. Finally, for some traits, humans and mice may be more alike than for others, which can additionally be aggravated by factors such as species-specific compensatory mechanisms or interactions with the environment. Regardless of the differing strength, however, we observed enrichment for a variety of the studied traits, supporting the general applicability of our approach.

Advantages of GenToS include its usefulness in settings where the sample size of subsequent GWAS cannot be increased easily, such as for rare diseases, or when replication studies may not be available. Further, the method can be extended to use additional evidence as input: although we used candidate gene input lists derived from murine phenotypes in this report, in principle any other candidate gene list could be used, such as candidate genes implicated by expression quantitative trait locus studies, candidate genes arising from GWAS carried out in other model organisms such as in the report of Wang *et al*. [18], or genes underlying monogenic human diseases. In support of the latter, many of the associations found with GenToS were already linked to human monogenic diseases in OMIM, supporting a model in which rare mutations of large effect and common variants of small effect in the same set of genes give rise to a continuum of a given human phenotype.

Some limitations of our approach warrant discussion: firstly, the performance of the method is influenced by the completeness of the candidate gene input lists. Although the work of the Jackson Lab and other groups has resulted in an impressively comprehensive and systematic resource of genetically manipulated and phenotyped mice, animal models were only available for 11,500 out of >25,000 murine genes at the time of our study. Because of issues such as early lethality or structurally complicated genomic regions that contain overlapping genes or are difficult to manipulate, the resource will likely never become complete. Together with the difficulty of quantifying some murine phenotypes, as discussed above, this may introduce misclassification that should bias any observed results towards the null. Another limitation is the inherent restriction to the available data when using posted GWAS summary results. For example, the conduct of approximate conditional analyses using the GWAS summary results would have been desirable to identify the presence of independent bone mineral density-associated SNPs in the *HOX* gene cluster, because murine phenotypes are observed for several of the genes in this cluster. However, this was not possible because the GEFOS Consortium did not make the estimated effect sizes required for these analyses publicly available. In addition, current GWAS are typically restricted to the evaluation of common genetic variants, and are therefore likely to miss association signals for rare variants of large effect. Future extensions of GWAS efforts and the continuing completion of the underlying murine MGI database will therefore likely result in further improvements of our findings.

In conclusion, GenToS is a flexible, freely available and user-friendly tool to incorporate external information in order to identify trait-associated SNPs in candidate genes that do not necessarily meet genome-wide significance in human GWAS studies. It allows for performing an analysis within minutes on a standard personal computer without any special requirements.

## Methods

### Generation of candidate gene input lists

Candidate genes, which when impaired cause skeletal phenotypes in mice, were selected by searching the Mouse Genome Informatics (MGI) resource [15]. MGI is the primary international database for laboratory mice. All phenotypes in MGI are categorized based on the Mammalian Phenotype (MP) ontology and emerge as a result of different genetic models, including targeted knockout animals, chemically induced (ENU) and spontaneous mutations. For this project, murine phenotypes were selected for their biomedical relevance regarding the evaluated traits for which GWAS data were publicly available, and downloaded from the MP ontology of MGI (http://www.informatics.jax.org/searches/MP_form.shtml) in March of 2015 for skeletal candidate gene lists and in June of 2015 for the glucose, insulin, systolic blood pressure and diabetes candidate gene lists (Table 3). For genes on each candidate gene list, human orthologs were selected using the Human-Mouse: Disease Connection [http://www.informatics.jax.org/humanDisease.html]. Genes with no ortholog in humans were filtered out; no other filtering criteria were used. The number of genes provided for each candidate gene list in this report represents the number of genes per list after translation to the human ortholog, the entry point for the use of GenTos.

### Genome-wide association study datasets

GenToS was applied to different publicly available datasets of GWAS summary statistics: 1. The **GEFOS** (GEnetic Factors for Osteoporosis) Consortium [19,20] is an international consortium investigating the genetic basis of osteoporosis. The datasets used in this report originated from the discovery step of two meta-analyses of GWAS summary statistics from different studies of European and East Asian ancestry that examined associations between genotyped and HapMap imputed single nucleotide polymorphisms and bone mineral density of the lumber spine (LSBMD; 32,000 individuals) and femoral neck (FNBMD; 33,000 individuals). 2. In the **MAGIC** (Meta-Analyses of Glucose and Insulin-related traits Consortium) [25] Consortium, international investigators investigate genetic influences on glucose metabolism. The datasets used in this report originated from discovery meta-analyses of fasting insulin (38,000 individuals) and fasting glucose (46,000 individuals) measured in non-diabetic individuals of European ancestry. 3. The **DIAGRAM** (DIAbetes Genetics Replication And Meta-analysis) [22] Consortium is a group of researchers aiming to characterize the genetic basis of type 2 diabetes. The datasets used in this report originated from the MAGIC discovery meta-analysis of type 2 diabetes (12,000 cases and 57,000 controls). 4. In the **ICBP** (International Consortium for Blood Pressure) [23,24], international investigators aim to understand the genetic underpinnings of blood pressure. The datasets used in this report originated from meta-analyses of genetic associations between SNPs and systolic blood pressure (SBP) among 74,000 participants of European ancestry. All datasets were downloaded from the respective consortium websites. Associations with human phenotypes were always evaluated accounting for the number of independent SNPs across all genes in a given candidate gene input list (see below).

Prior to use with GenToS, GWAS meta-analysis summary datasets were lifted over from hg18 to hg19 using the UCSC lift-over tool, and were subsequently converted into a sqlite3 database using a custom script.

### GenToS

GenToS involves three different steps to identify loci from a given GWAS summary statistics file with p-values below a computed or user-specified significance threshold. To do so, GenToS requires the GWAS summary statistics file as well as a file containing a single or several candidate genes, an input list. In this report, this candidate gene list contained genes that cause specific phenotypes in genetically manipulated mice (see above).

1. Definition of region

For each gene of a given candidate gene input list, GenToS extracts the starting and ending genomic coordinates of that gene in order to determine the regions of interest. The positions are extracted from a pre-computed database containing the starting and ending positions of all genes. The positions in this database are based on the longest transcript for each gene (see pre-computed databases, below). Genes with ambiguous starting or ending positions, mostly due to mapping to different chromosomes, were excluded. In addition, for the GWAS traits evaluated in this report, genes mapping to human gonosomes were excluded because no GWAS summary statistics were available for X- or Y-chromosomal SNPs. For each remaining gene from the candidate gene input list, a user-defined flanking region, by default 10kb upstream and downstream of the gene’s starting and ending position, is added to the extracted positions to determine the gene region to be used within GenToS (Fig 1A). Thus, the evaluated gene regions contain exons, introns and proximal regulatory elements such as promoters for each gene.

2. Calculation of the statistical significance threshold

The default method to define the significance threshold for a given invocation of GenToS is based on a Bonferroni correction of a type I error probability of 0.05 for the number of independent SNPs in a given gene region. GenToS extracts the number of independent SNPs from a pre-computed database of independent SNPs based on the 1000 Genomes Project phase 1 version 3 (see below). The genetic ancestry of the reference population used to derive the number of independent SNPs (EUR, AFR, ASN and ALL) can be chosen by an option. For candidate gene input lists containing more than one gene, an option can be used that uses the sum of all independent SNPs across all gene regions to determine the Bonferroni-corrected significance threshold, in order to account for the testing of multiple genes. As additional methods to determine the statistical significance threshold, options for setting a user-defined threshold as well as a FDR-based threshold are implemented (Fig 1B).

3. Extraction of significantly associated SNPs from GWAS

As a final step, GenToS searches the specified GWAS summary data file for SNPs within the defined gene regions with association p-values lower than the determined significance threshold. If present, summary statistics for such SNPs are annotated to the gene of interest and written to a results file. Consequently, the results file contains all information present in the input GWAS summary file, along with gene mapping information (Fig 1C).

Subsequent to this three-step procedure an optional yet recommended step is implemented to evaluate whether there is significant enrichment of the number of detected association signals for the genes contained in the candidate gene input list compared to the number of detected associations expected by chance alone. Assessment of enrichment can be carried out by visual comparison to the null distribution, which is generated based on the number of significant genes identified in GWAS data based on the iterative evaluation of randomly drawn input gene lists (2,000 iterations by default) that contain an equal number of genes as the evaluated candidate gene input. The number of 2,000 iterations was chosen as a compromise between computational time and sufficient precision. Because each of the 2,000 iterations generates an input gene list of the same number but different genes (i.e. randomly drawn) the calculation of the number of independent SNPs across each list followed by a Bonferroni correction procedure is carried out for each draw. This procedure accounts for the different size and linkage disequilibrium structure of genes within and across lists, and represents a time consuming yet reliable method to derive a null distribution. Another option to assess enrichment is a similar graphical representation based on a binomial distribution, where the probability p of a significant association is estimated by the proportion of the total number of genes with GWAS association signals below the calculated significance threshold for the given candidate gene input list among the total number of genes in the gene database and n is the total number of genes on the candidate gene input list. The probability of observing as many or more significant genes x is then estimated using a complementary cumulative binomial distribution (enrichment p-value).

$$P\left(X\geq x\right)\;=\;1-\left(\overset{x-1}{\underset{k\;=\;0}{\sum }}\left(\begin{array}{c} n\\ k \end{array}\right)p^{k}{\left(1-p\right)}^{n-k}\right)$$

Genes implicated by GenToS were further investigated by annotating them using the Online Mendelian Inheritance in Man (OMIM) resource as well as the annotation program SNiPA [29].

### Pre-computed databases

In order to run GenToS, two databases, one containing the genes and their positions in the genome and the other containing independent SNPs across the genome were pre-computed.

For the gene database, all RefSeq genes (table refFlat) were downloaded from the UCSC homepage using build GRCh37/hg19 coordinates [30]. In a subsequent processing step, the longest transcript for each gene was retained. Only genes of unambiguous mapping and for which starting and ending position were not mapping onto different chromosomes were extracted and added to the database for a total of 25,230 entries.

The independent SNPs for the SNP database were pre-computed based on the 1000 Genomes project phase 1 version 3 data using plink (version 1.90b2) [31] (options—indep-pairwise 50 5 0.2 and—maf 0.01). The computation was carried out chromosome-wise and added to the SNP database, each chromosome in a different table.

### Algorithm Information

GenToS is a command line based tool implemented in java to run on a Linux based desktop PC. For a typical analysis, as provided in this paper, a single core processor with 5 GB of memory is required. An implementation of GenToS including examples can be downloaded at https://github.com/genepi-freiburg/gentos. Databases used in GenToS are based on SQlite and produced using a custom perl script also available at github. For the generation of custom databases, database specifications are provided in the GenToS help.

## Supporting Information

S1 Fig QQ-plots of the number of observed significant genes under the null hypothesis comparing random draws of input gene lists and simulated draws based on a binomial experiment for the number of genes contained on the candidate gene list “skeleton morphology”. (PDF)

S2 Fig QQ-plots of the number of observed significant genes under the null hypothesis comparing random draws of input gene lists and simulated draws based on a binomial experiment for the number of genes contained on the candidate gene list “abnormal bone mineralization”. (PDF)

S3 Fig Enrichment of significant SNP associations in human GWAS of lumbar spine bone mineral density for a candidate gene input list that contains genes underlying skeletal phenotypes in mice. (PDF)

S4 Fig Regional association plots of loci associated with human bone mineral density phenotypes, which were not implicated as causal genes or not associated at genome-wide significance in previous GWAS. (PDF)

S5 Fig Enrichment of significant SNP associations in human GWAS of type 2 diabetes for genes causing impaired glucose handling in mice. (PDF)

S6 Fig Enrichment of significant SNP associations in human GWAS of blood pressure phenotypes for genes causing corresponding traits in mice. (PDF)

S7 Fig Enrichment of significant SNP associations in human GWAS of fasting insulin concentrations for genes causing impaired insulin levels in mice. (PDF)

S8 Fig Enrichment of significant SNP associations in human GWAS of fasting glucose concentrations for genes causing impaired glucose levels in mice. (PDF)

S1 Table Genes identified by GenToS with significant SNP associations with diabetes, glycemic measures and blood pressure measurements. (PDF)

## References

[1] Visscher PM, Brown MA, McCarthy MI, Yang J (2012) Five years of GWAS discovery. Am J Hum Genet 90: 7–24. 10.1016/j.ajhg.2011.11.029 22243964 PMC3257326

[2] Welter D, MacArthur J, Morales J, Burdett T, Hall P, Junkins H, et al (2014) The NHGRI GWAS Catalog, a curated resource of SNP-trait associations. Nucleic Acids Res 42: D1001–1006. 10.1093/nar/gkt1229 24316577 PMC3965119

[3] Pe'er I, Yelensky R, Altshuler D, Daly MJ (2008) Estimation of the multiple testing burden for genomewide association studies of nearly all common variants. Genet Epidemiol 32: 381–385. 10.1002/gepi.20303 18348202

[4] Wuttke M, Wong CS, Wuhl E, Epting D, Luo L, Hoppmann A, et al (2015) Genetic loci associated with renal function measures and chronic kidney disease in children: the Pediatric Investigation for Genetic Factors Linked with Renal Progression Consortium. Nephrol Dial Transplant. 10.1093/ndt/gfv342 PMC4829056 26420894

[5] McCarthy MI, Abecasis GR, Cardon LR, Goldstein DB, Little J, Ioannidis JP, et al (2008) Genome-wide association studies for complex traits: consensus, uncertainty and challenges. Nat Rev Genet 9: 356–369. 10.1038/nrg2344 18398418

[6] Raychaudhuri S, Plenge RM, Rossin EJ, Ng AC, International Schizophrenia C, Purcell SM, et al (2009) Identifying relationships among genomic disease regions: predicting genes at pathogenic SNP associations and rare deletions. PLoS Genet 5: e1000534 10.1371/journal.pgen.1000534 19557189 PMC2694358

[7] Lee I, Blom UM, Wang PI, Shim JE, Marcotte EM (2011) Prioritizing candidate disease genes by network-based boosting of genome-wide association data. Genome Res 21: 1109–1121. 10.1101/gr.118992.110 21536720 PMC3129253

[8] Moreau Y, Tranchevent LC (2012) Computational tools for prioritizing candidate genes: boosting disease gene discovery. Nat Rev Genet 13: 523–536. 10.1038/nrg3253 22751426

[9] Deo RC, Musso G, Tasan M, Tang P, Poon A, Yuan C, et al (2014) Prioritizing causal disease genes using unbiased genomic features. Genome Biol 15: 534 10.1186/s13059-014-0534-8 25633252 PMC4279789

[10] Pers TH, Dworzynski P, Thomas CE, Lage K, Brunak S (2013) MetaRanker 2.0: a web server for prioritization of genetic variation data. Nucleic Acids Res 41: W104–108. 10.1093/nar/gkt387 23703204 PMC3692047

[11] Pers TH, Karjalainen JM, Chan Y, Westra HJ, Wood AR, Yang J, et al (2015) Biological interpretation of genome-wide association studies using predicted gene functions. Nat Commun 6: 5890 10.1038/ncomms6890 25597830 PMC4420238

[12] Butkiewicz M, Cooke Bailey JN, Frase A, Dudek S, Yaspan BL, Ritchie MD, et al (2016) Pathway analysis by randomization incorporating structure-PARIS: an update. Bioinformatics 32: 2361–2363. 10.1093/bioinformatics/btw130 27153576 PMC4965631

[13] Kanehisa M, Goto S, Sato Y, Kawashima M, Furumichi M, Tanabe M (2014) Data, information, knowledge and principle: back to metabolism in KEGG. Nucleic Acids Res 42: D199–205. 10.1093/nar/gkt1076 24214961 PMC3965122

[14] Gene Ontology C (2015) Gene Ontology Consortium: going forward. Nucleic Acids Res 43: D1049–1056. 10.1093/nar/gku1179 25428369 PMC4383973

[15] Eppig JT, Blake JA, Bult CJ, Kadin JA, Richardson JE, Mouse Genome Database G (2015) The Mouse Genome Database (MGD): facilitating mouse as a model for human biology and disease. Nucleic Acids Res 43: D726–736. 10.1093/nar/gku967 25348401 PMC4384027

[16] Skarnes WC, Rosen B, West AP, Koutsourakis M, Bushell W, Iyer V, et al (2011) A conditional knockout resource for the genome-wide study of mouse gene function. Nature 474: 337–342. 10.1038/nature10163 21677750 PMC3572410

[17] Ashbrook DG, Williams RW, Lu L, Hager R (2015) A cross-species genetic analysis identifies candidate genes for mouse anxiety and human bipolar disorder. Front Behav Neurosci 9: 171 10.3389/fnbeh.2015.00171 26190982 PMC4486840

[18] Wang X, Pandey AK, Mulligan MK, Williams EG, Mozhui K, Li Z, et al (2016) Joint mouse-human phenome-wide association to test gene function and disease risk. Nat Commun 7: 10464 10.1038/ncomms10464 26833085 PMC4740880

[19] Estrada K, Styrkarsdottir U, Evangelou E, Hsu YH, Duncan EL, Ntzani EE, et al (2012) Genome-wide meta-analysis identifies 56 bone mineral density loci and reveals 14 loci associated with risk of fracture. Nat Genet 44: 491–501. 10.1038/ng.2249 22504420 PMC3338864

[20] Rivadeneira F, Styrkarsdottir U, Estrada K, Halldorsson BV, Hsu YH, Richards JB, et al (2009) Twenty bone-mineral-density loci identified by large-scale meta-analysis of genome-wide association studies. Nat Genet 41: 1199–1206. 10.1038/ng.446 19801982 PMC2783489

[21] Zhang L, Choi HJ, Estrada K, Leo PJ, Li J, Pei YF, et al (2014) Multistage genome-wide association meta-analyses identified two new loci for bone mineral density. Hum Mol Genet 23: 1923–1933. 10.1093/hmg/ddt575 24249740 PMC3943521

[22] Morris AP, Voight BF, Teslovich TM, Ferreira T, Segre AV, Steinthorsdottir V, et al (2012) Large-scale association analysis provides insights into the genetic architecture and pathophysiology of type 2 diabetes. Nat Genet 44: 981–990. 10.1038/ng.2383 22885922 PMC3442244

[23] Wain LV, Verwoert GC, O'Reilly PF, Shi G, Johnson T, Johnson AD, et al (2011) Genome-wide association study identifies six new loci influencing pulse pressure and mean arterial pressure. Nat Genet 43: 1005–1011. 10.1038/ng.922 21909110 PMC3445021

[24] International Consortium for Blood Pressure Genome-Wide Association S, Ehret GB, Munroe PB, Rice KM, Bochud M, Johnson AD, et al (2011) Genetic variants in novel pathways influence blood pressure and cardiovascular disease risk. Nature 478: 103–109. 10.1038/nature10405 21909115 PMC3340926

[25] Dupuis J, Langenberg C, Prokopenko I, Saxena R, Soranzo N, Jackson AU, et al (2010) New genetic loci implicated in fasting glucose homeostasis and their impact on type 2 diabetes risk. Nat Genet 42: 105–116. 10.1038/ng.520 20081858 PMC3018764

[26] Wang K, Li M, Bucan M (2007) Pathway-based approaches for analysis of genomewide association studies. Am J Hum Genet 81: 1278–1283. 17966091 10.1086/522374 PMC2276352

[27] Lee PH, O'Dushlaine C, Thomas B, Purcell SM (2012) INRICH: interval-based enrichment analysis for genome-wide association studies. Bioinformatics 28: 1797–1799. 10.1093/bioinformatics/bts191 22513993 PMC3381960

[28] Segre AV, Consortium D, investigators M, Groop L, Mootha VK, Daly MJ, et al (2010) Common inherited variation in mitochondrial genes is not enriched for associations with type 2 diabetes or related glycemic traits. PLoS Genet 6. 10.1371/journal.pgen.1001058 PMC2920848 20714348

[29] Arnold M, Raffler J, Pfeufer A, Suhre K, Kastenmuller G (2015) SNiPA: an interactive, genetic variant-centered annotation browser. Bioinformatics 31: 1334–1336. 10.1093/bioinformatics/btu779 25431330 PMC4393511

[30] Kent WJ, Sugnet CW, Furey TS, Roskin KM, Pringle TH, Zahler AM, et al (2002) The human genome browser at UCSC. Genome Research 12: 996–1006. 12045153 10.1101/gr.229102 PMC186604

[31] Purcell S, Neale B, Todd-Brown K, Thomas L, Ferreira MA, Bender D, et al (2007) PLINK: a tool set for whole-genome association and population-based linkage analyses. Am J Hum Genet 81: 559–575. 17701901 10.1086/519795 PMC1950838

