# Supplementary figures and images for: GenToS: Use of Orthologous Gene Information to Prioritize Signals from Human GWAS
Source: PLoS One. 2016 Sep 9;11(9):e0162466. doi: 10.1371/journal.pone.0162466 (PMC5017755; doi:10.1371/journal.pone.0162466)

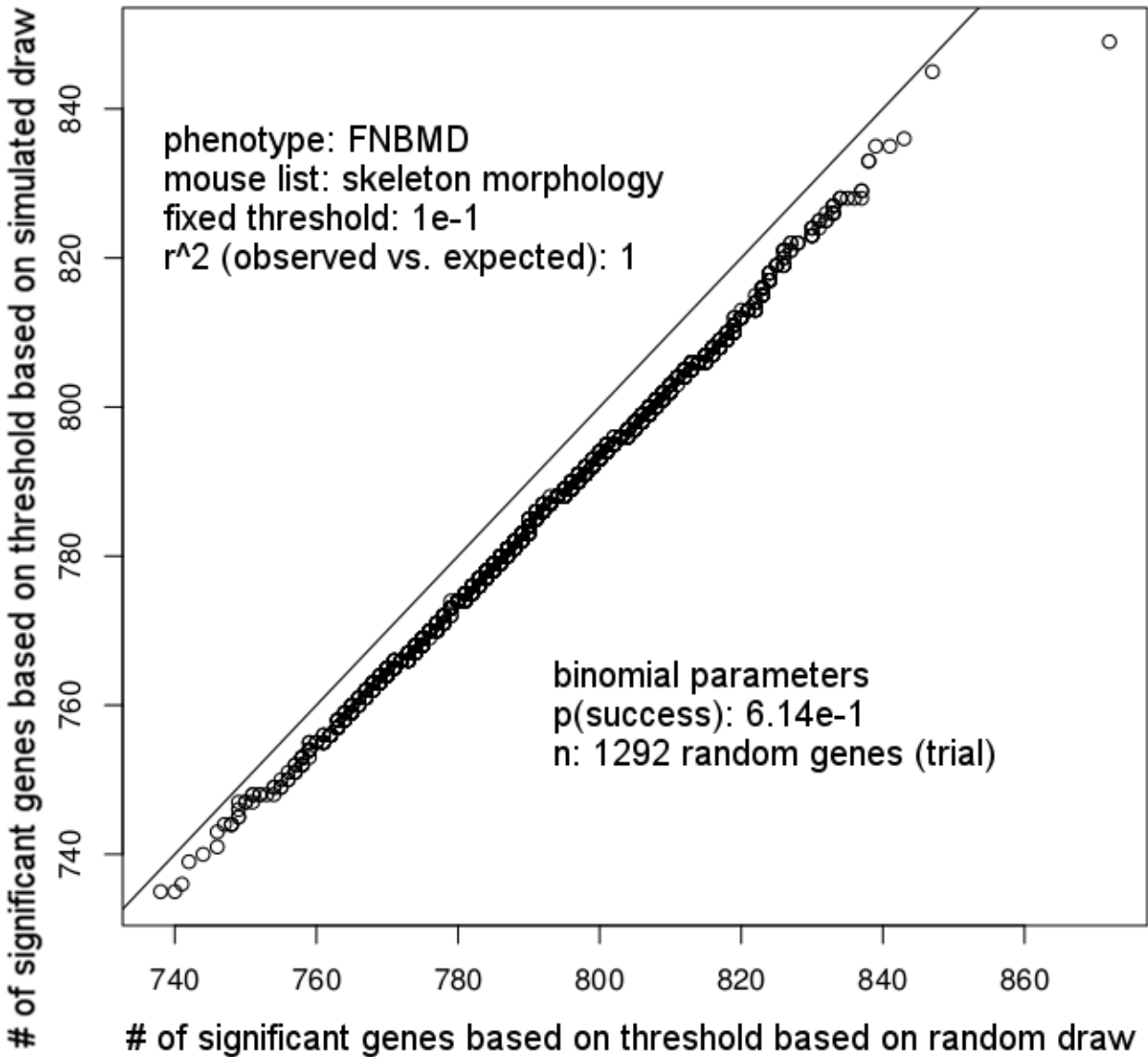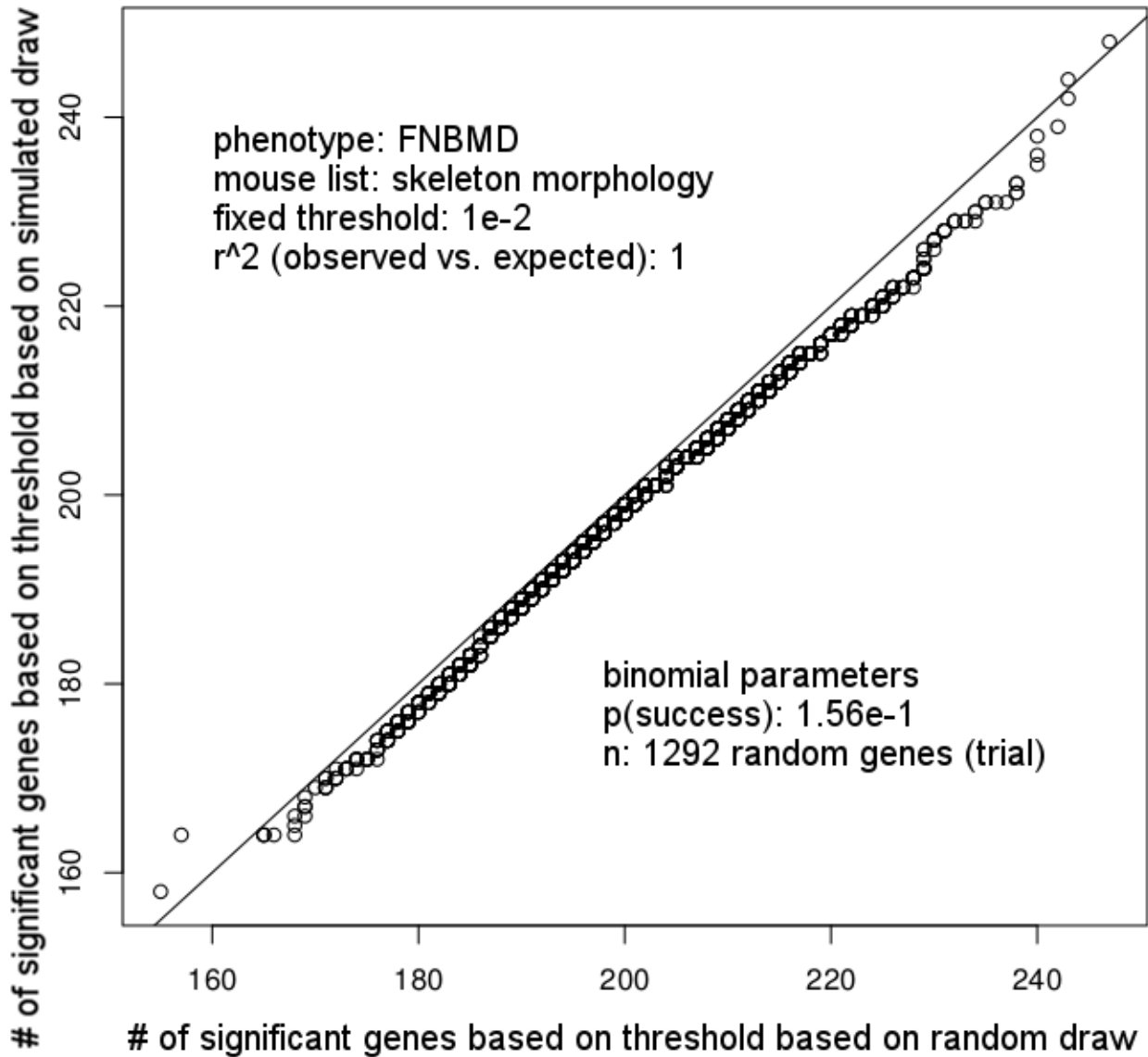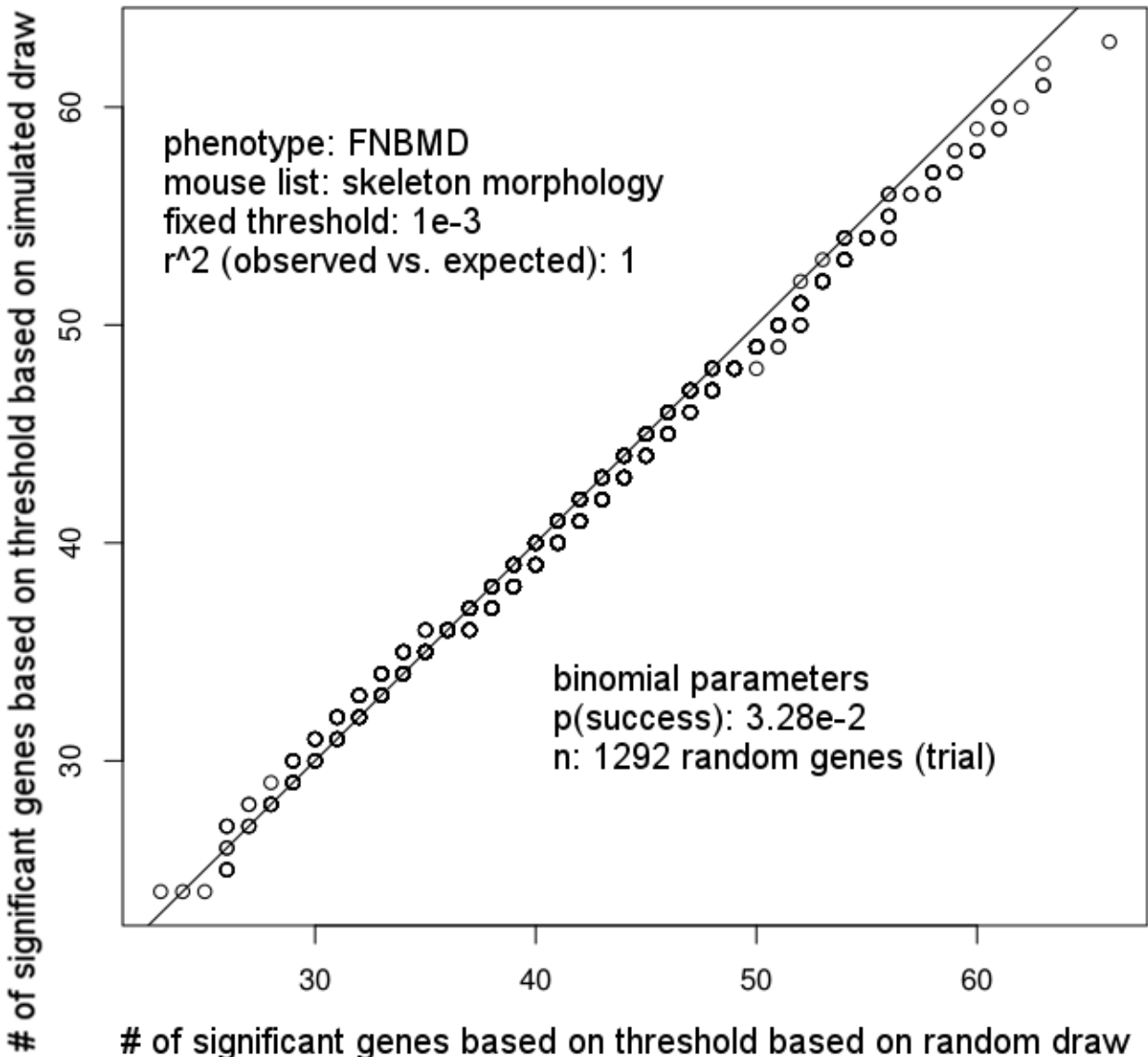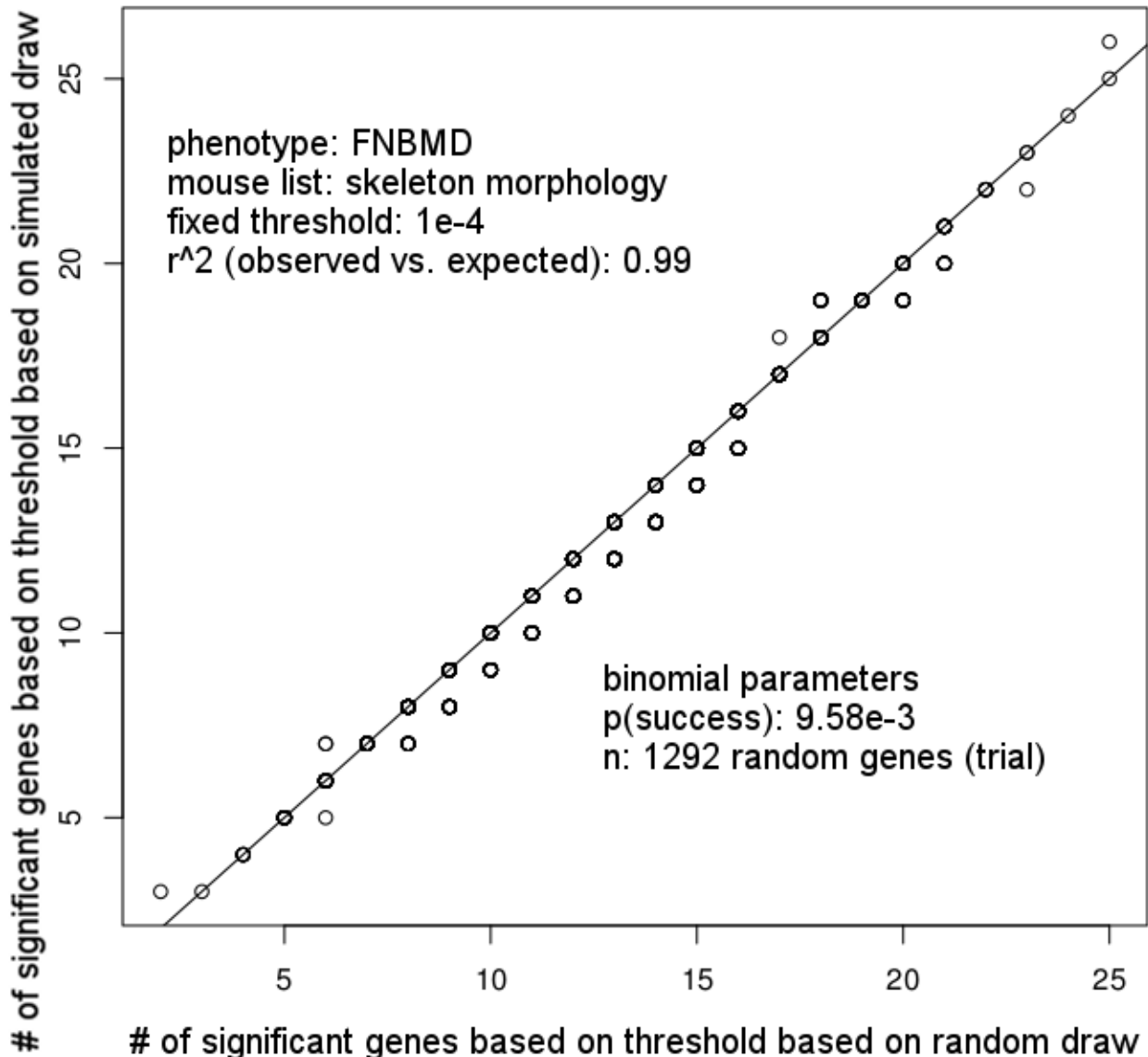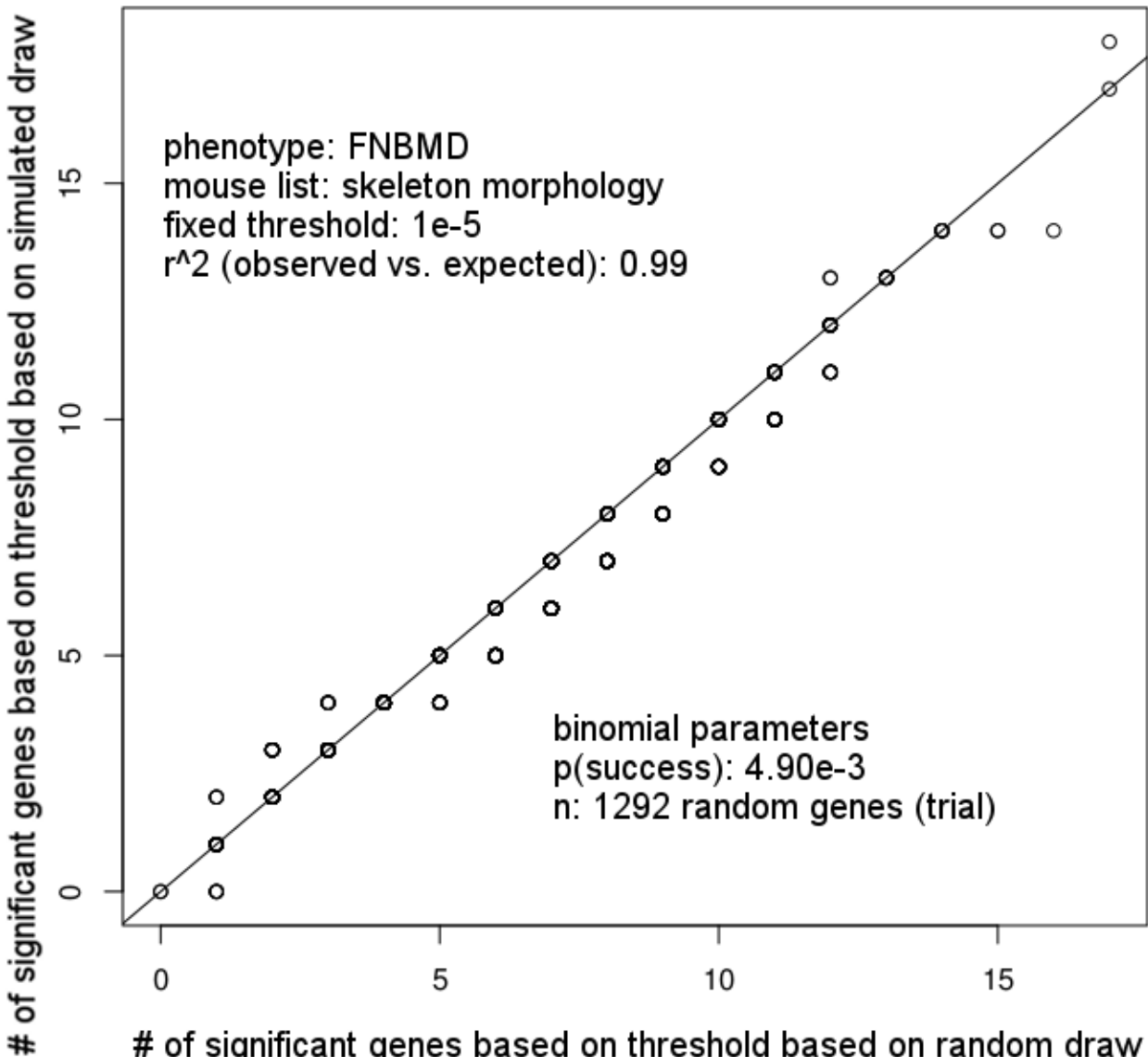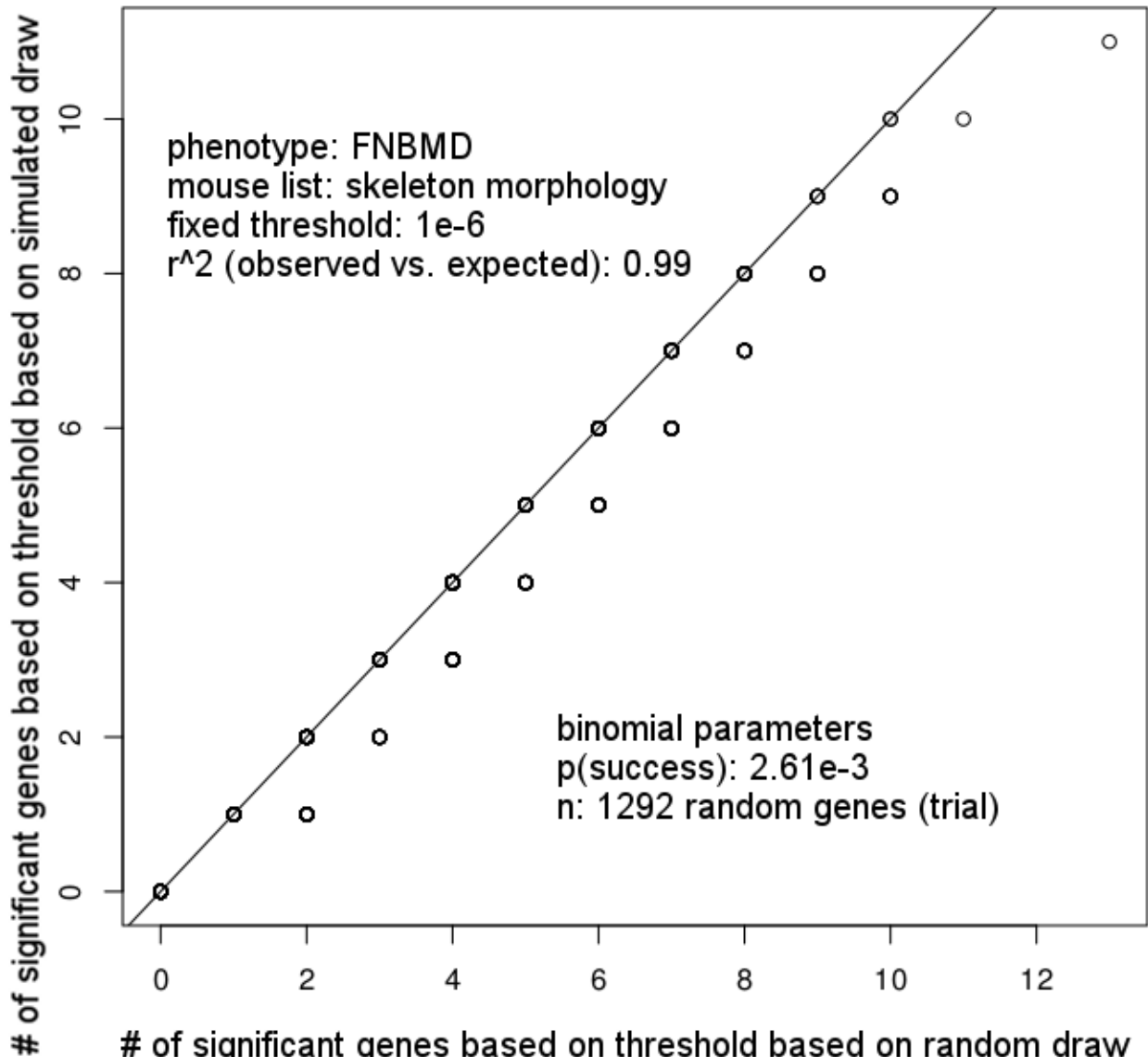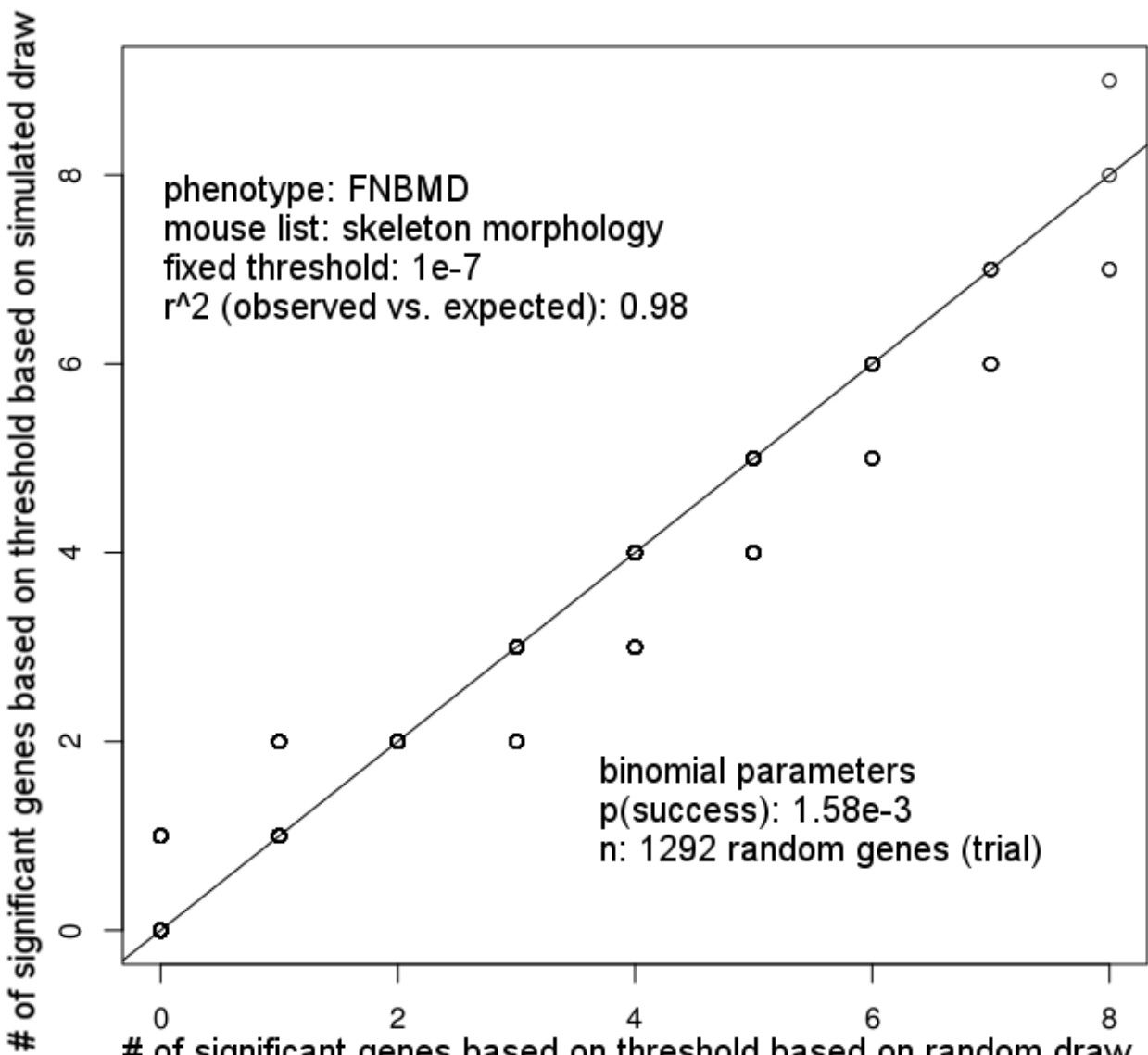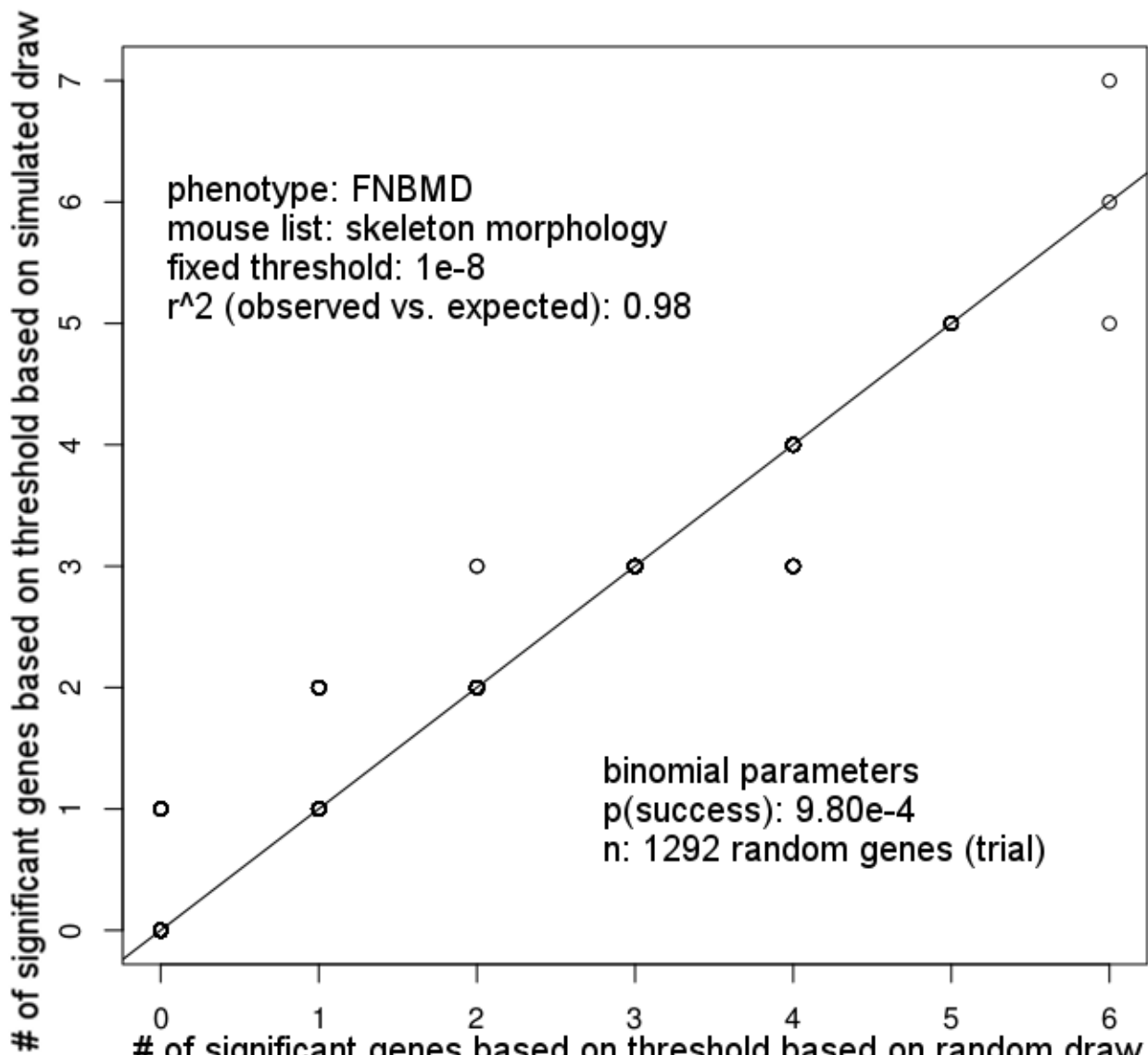

Supplement: S1 Fig — (PDF) [file pone.0162466.s001.pdf]

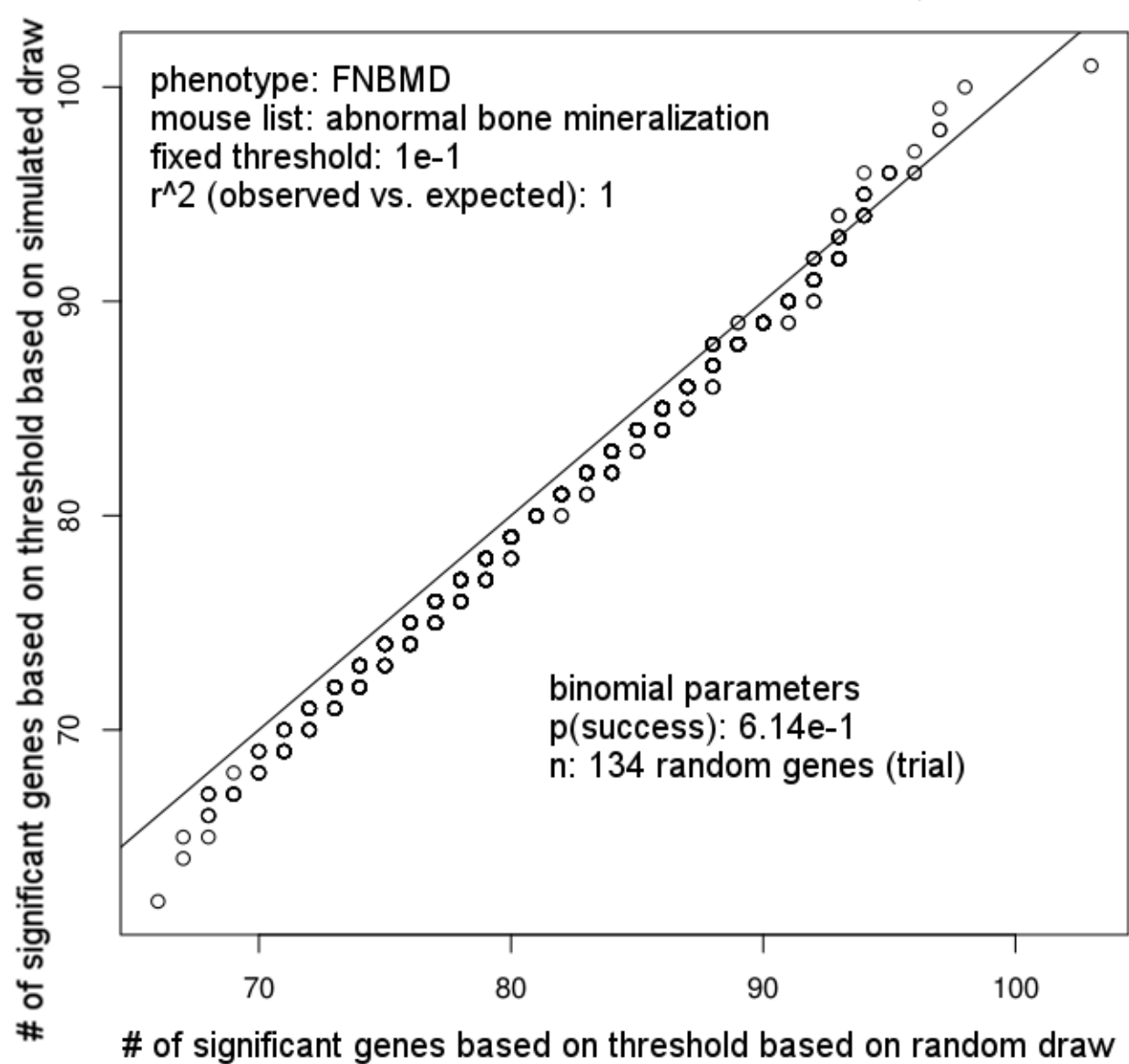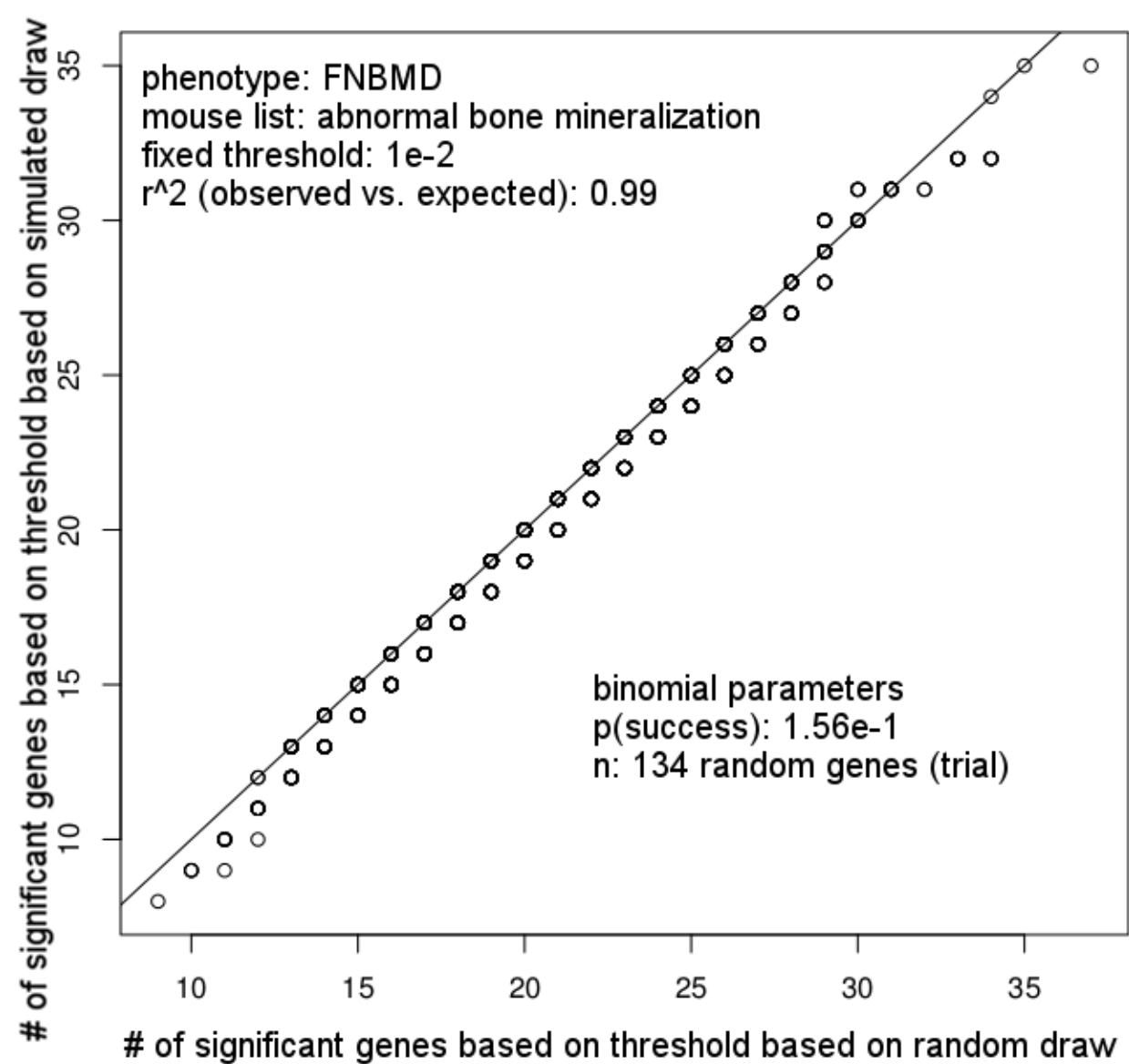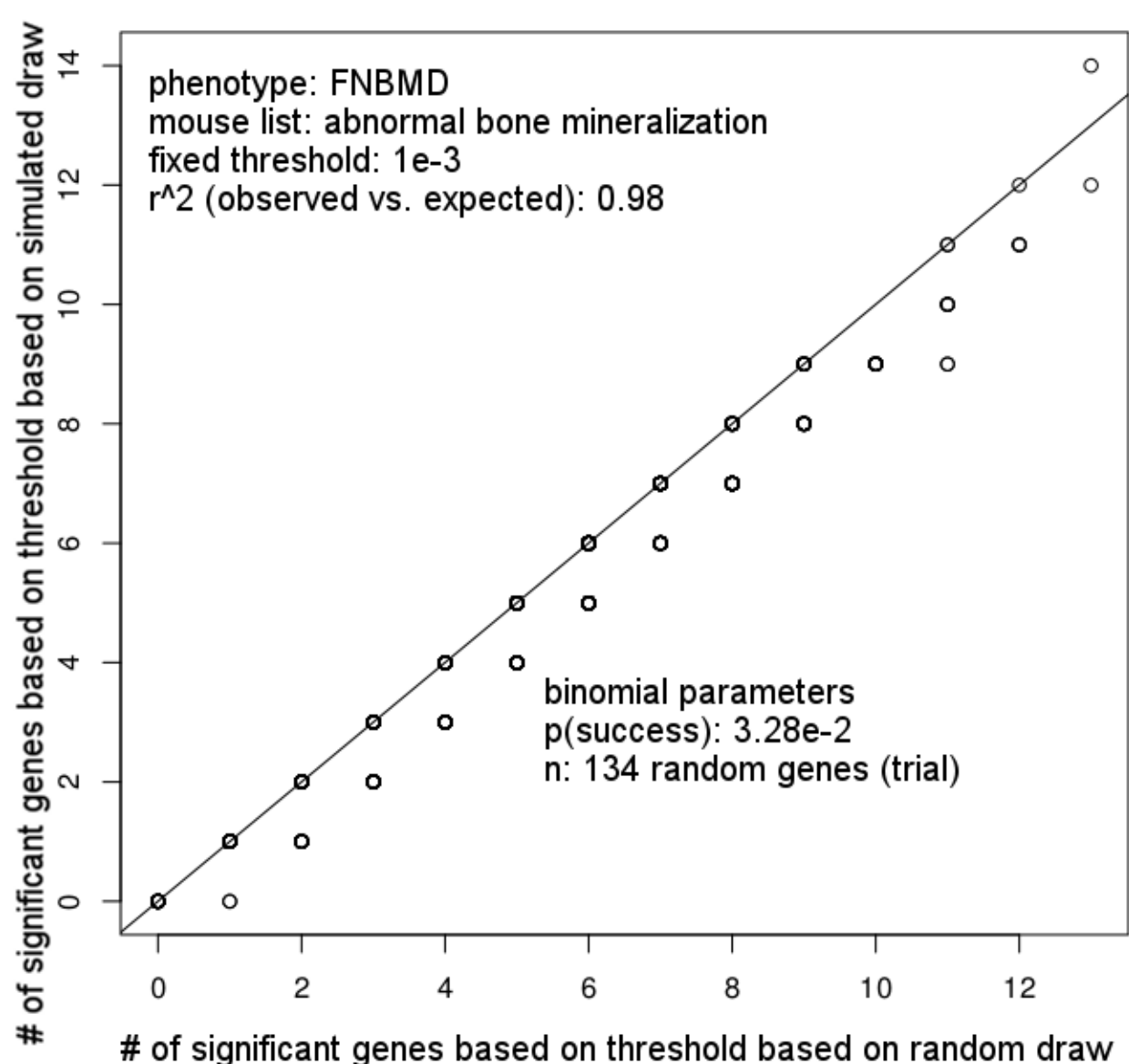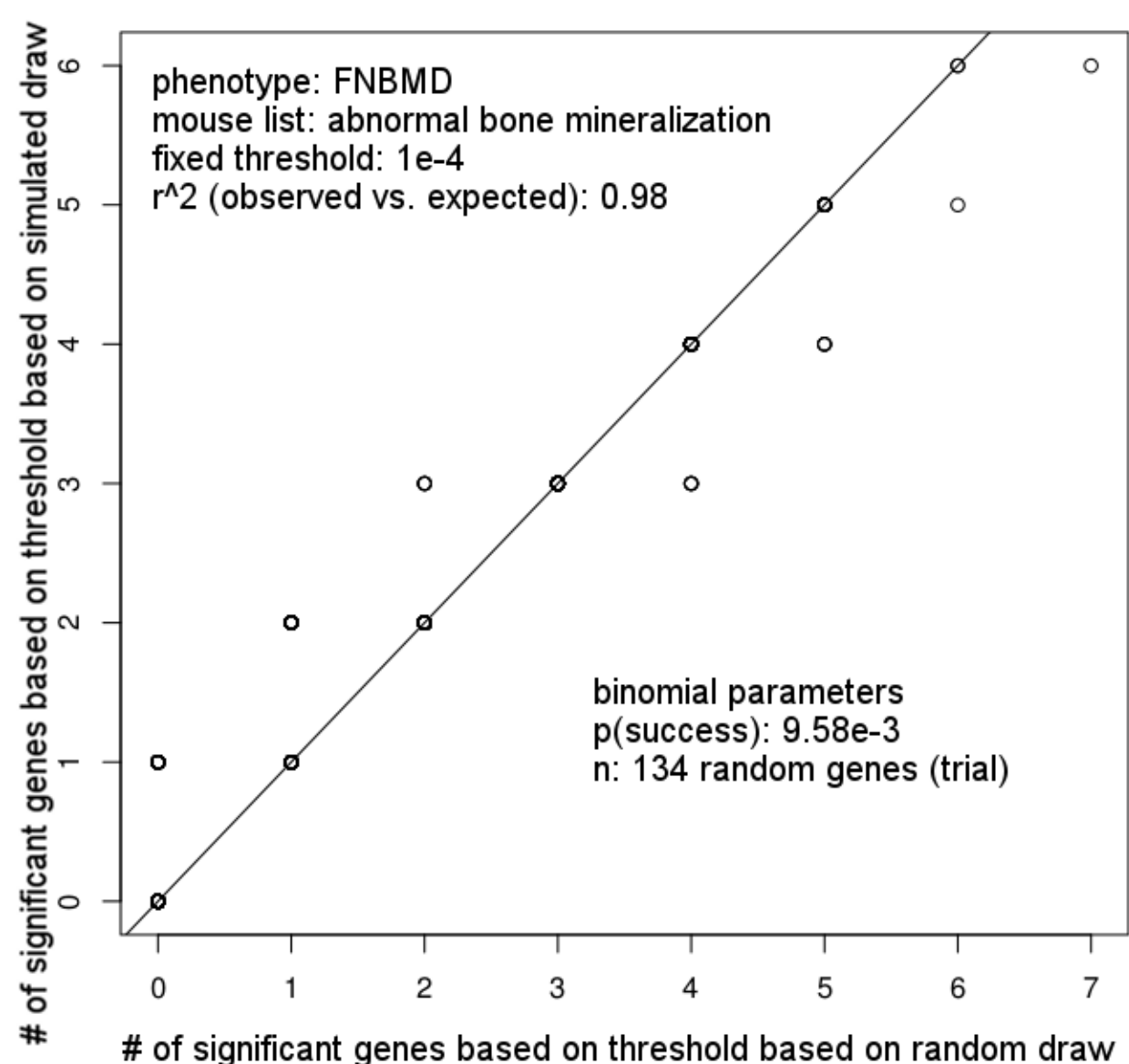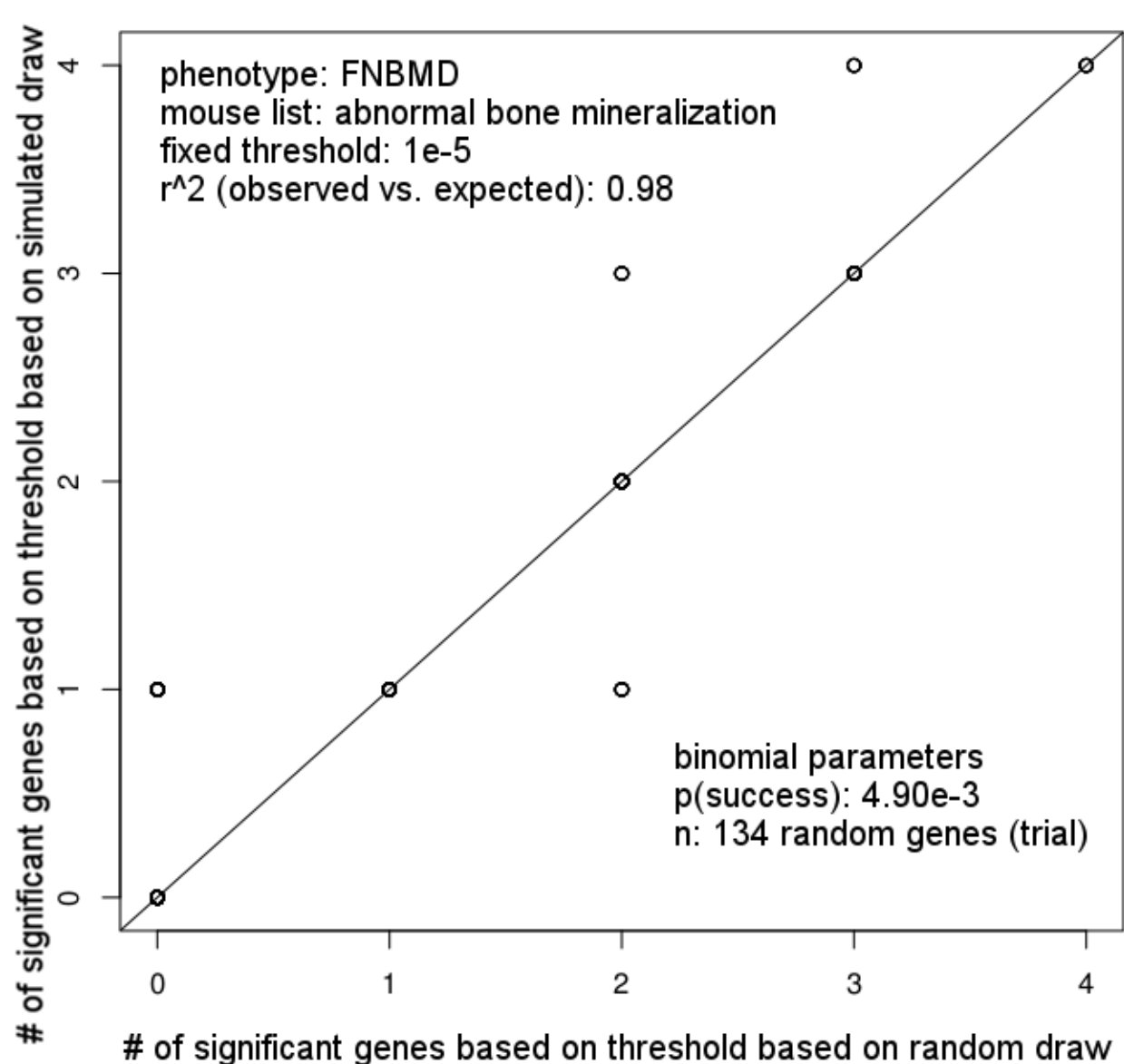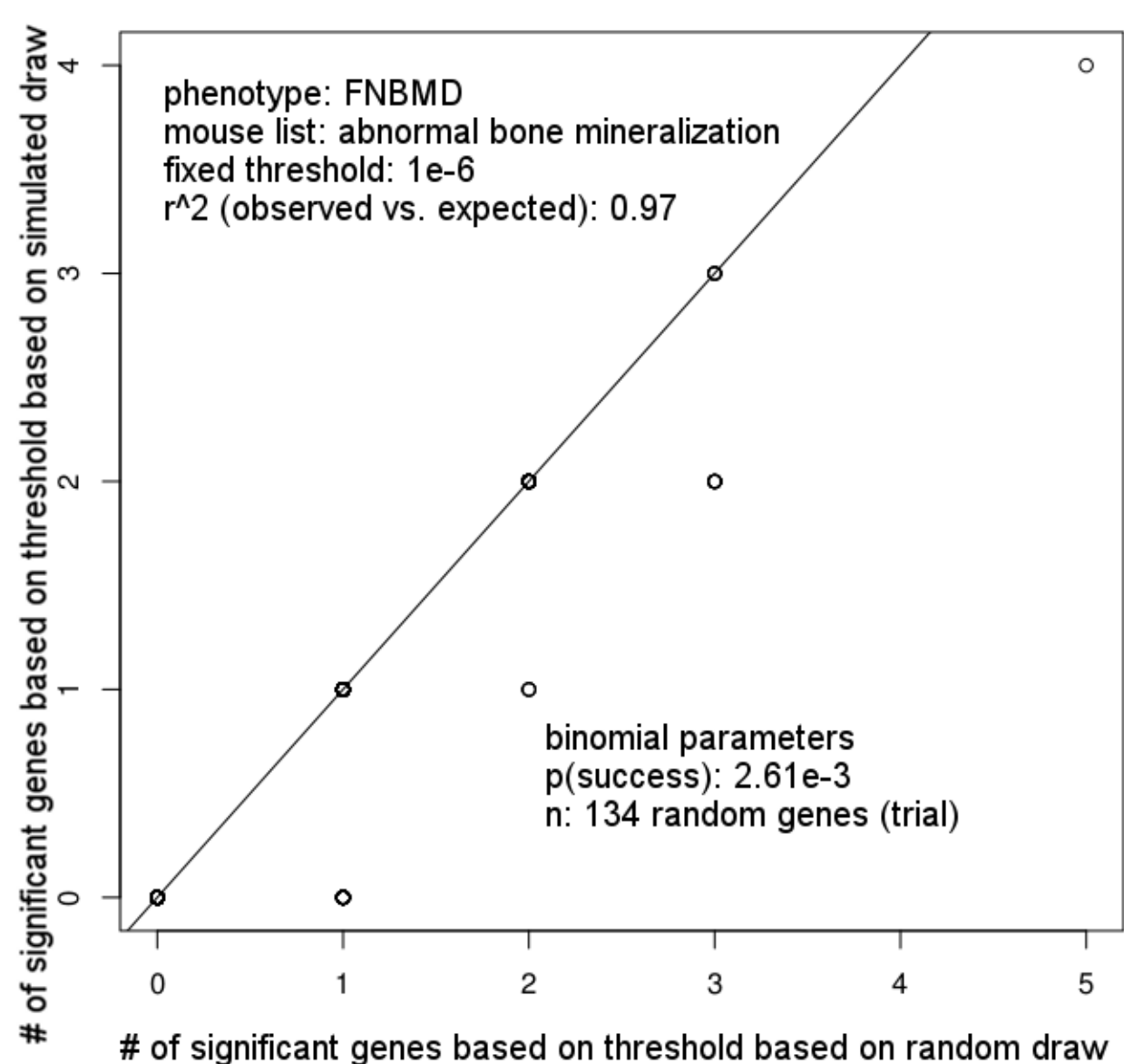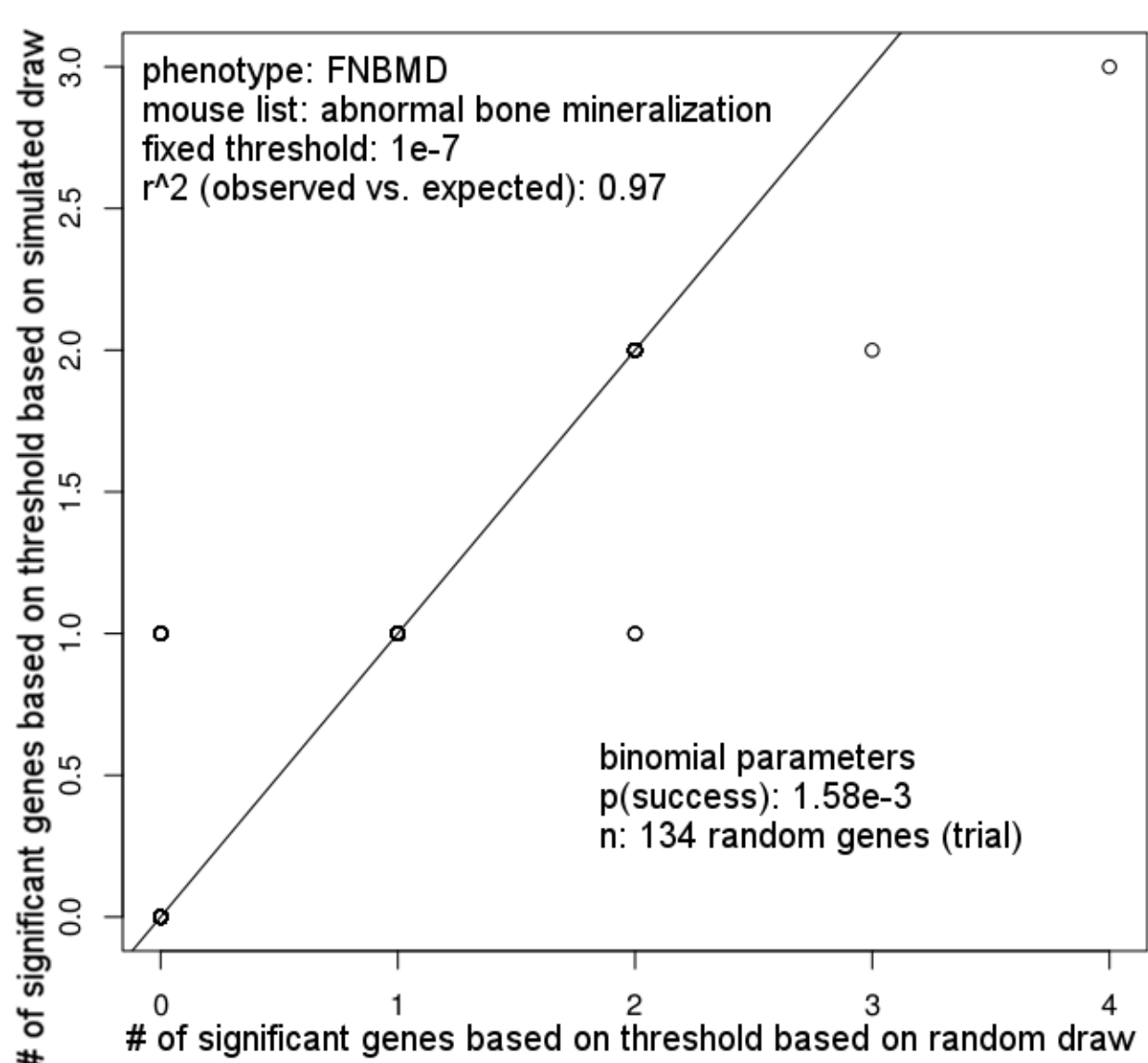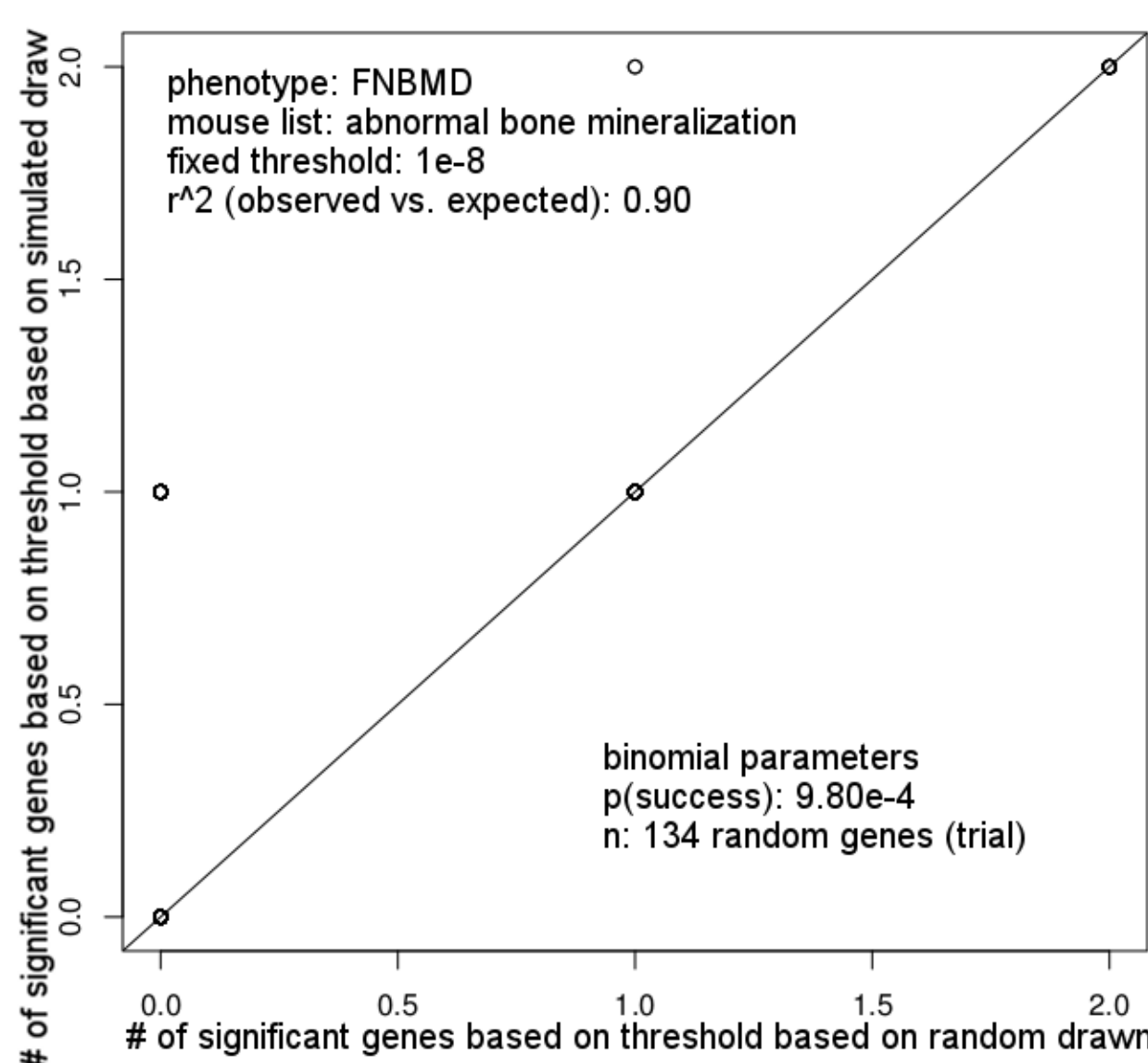

Supplement: S2 Fig — (PDF) [file pone.0162466.s002.pdf]

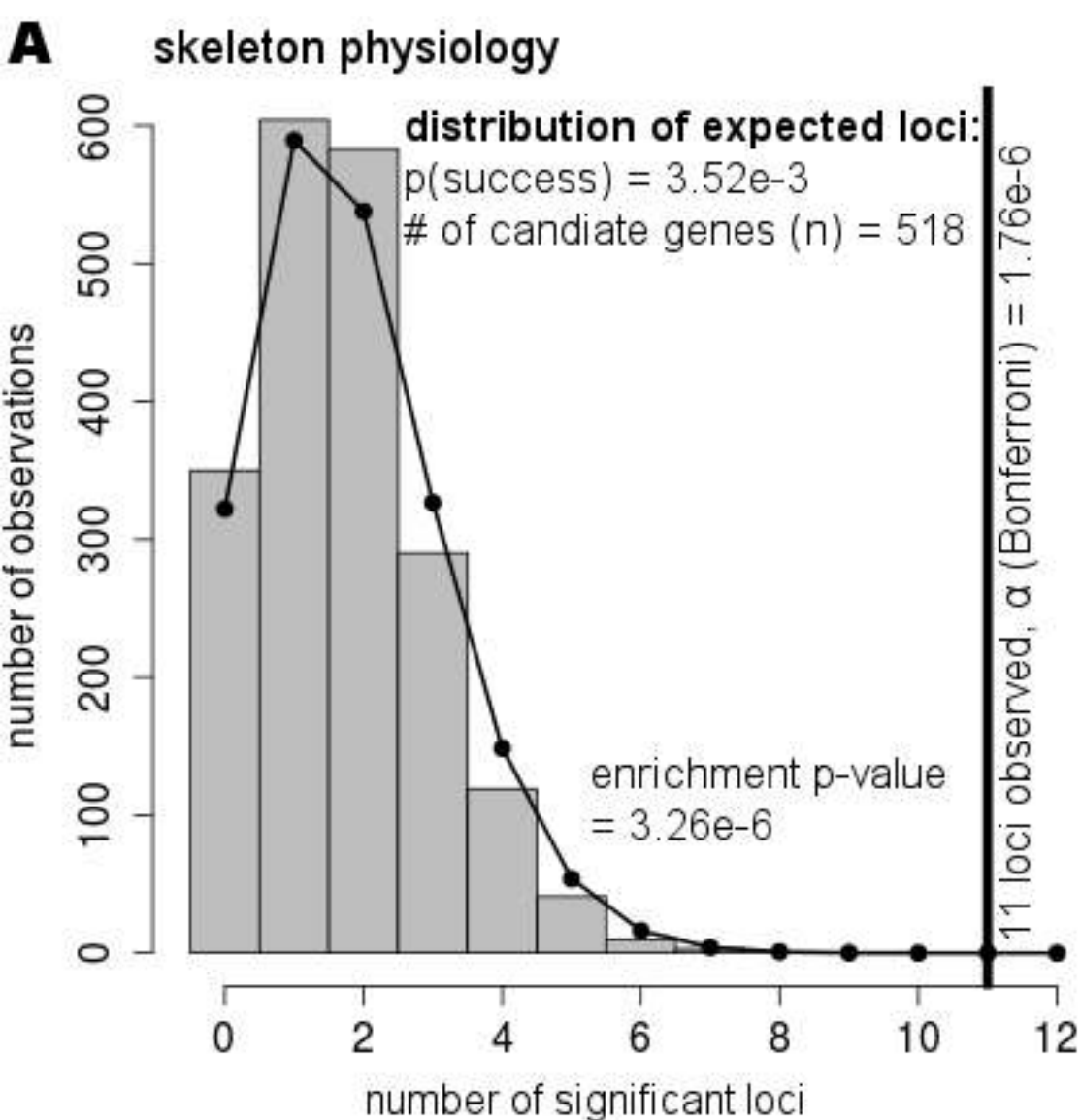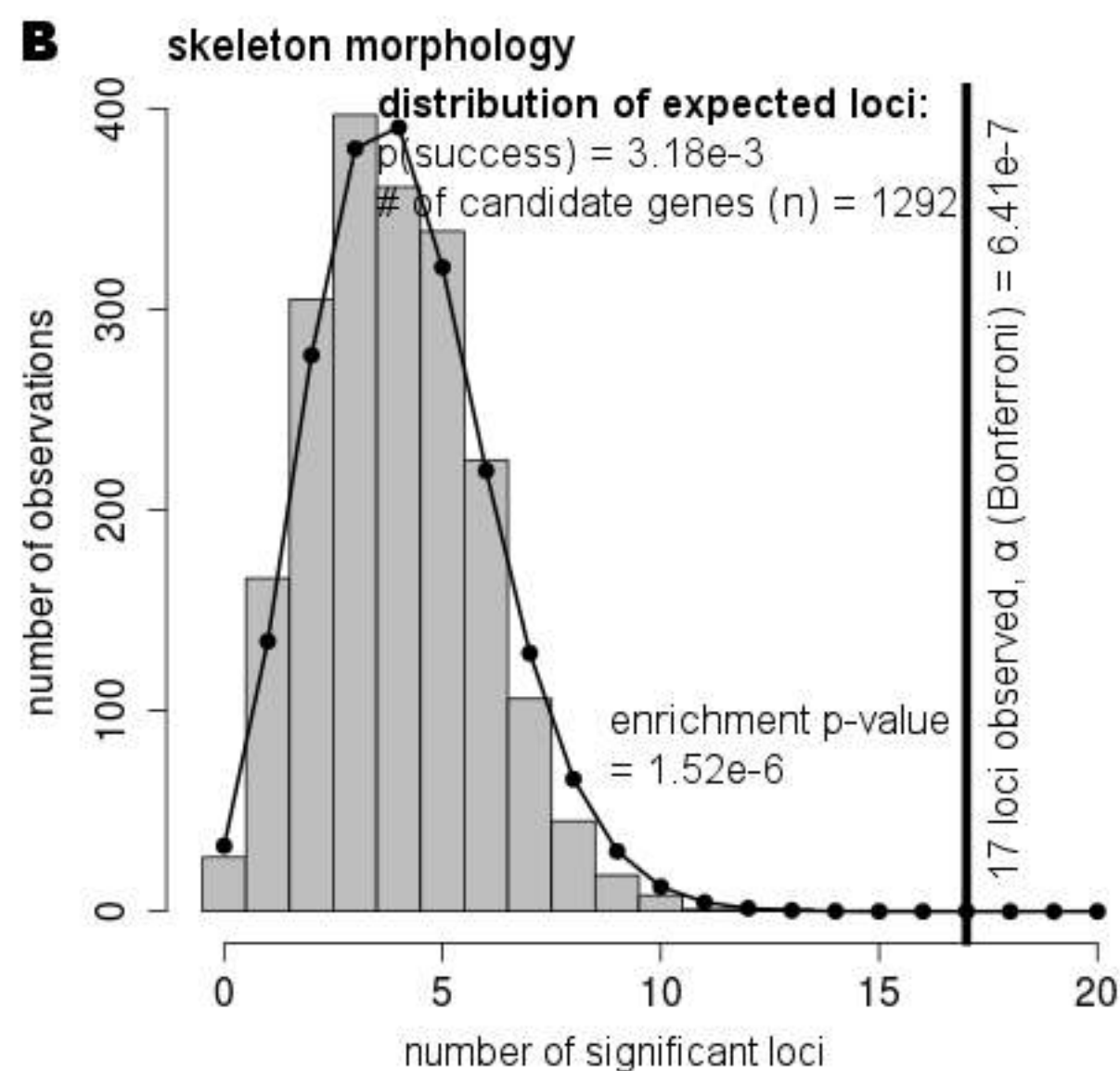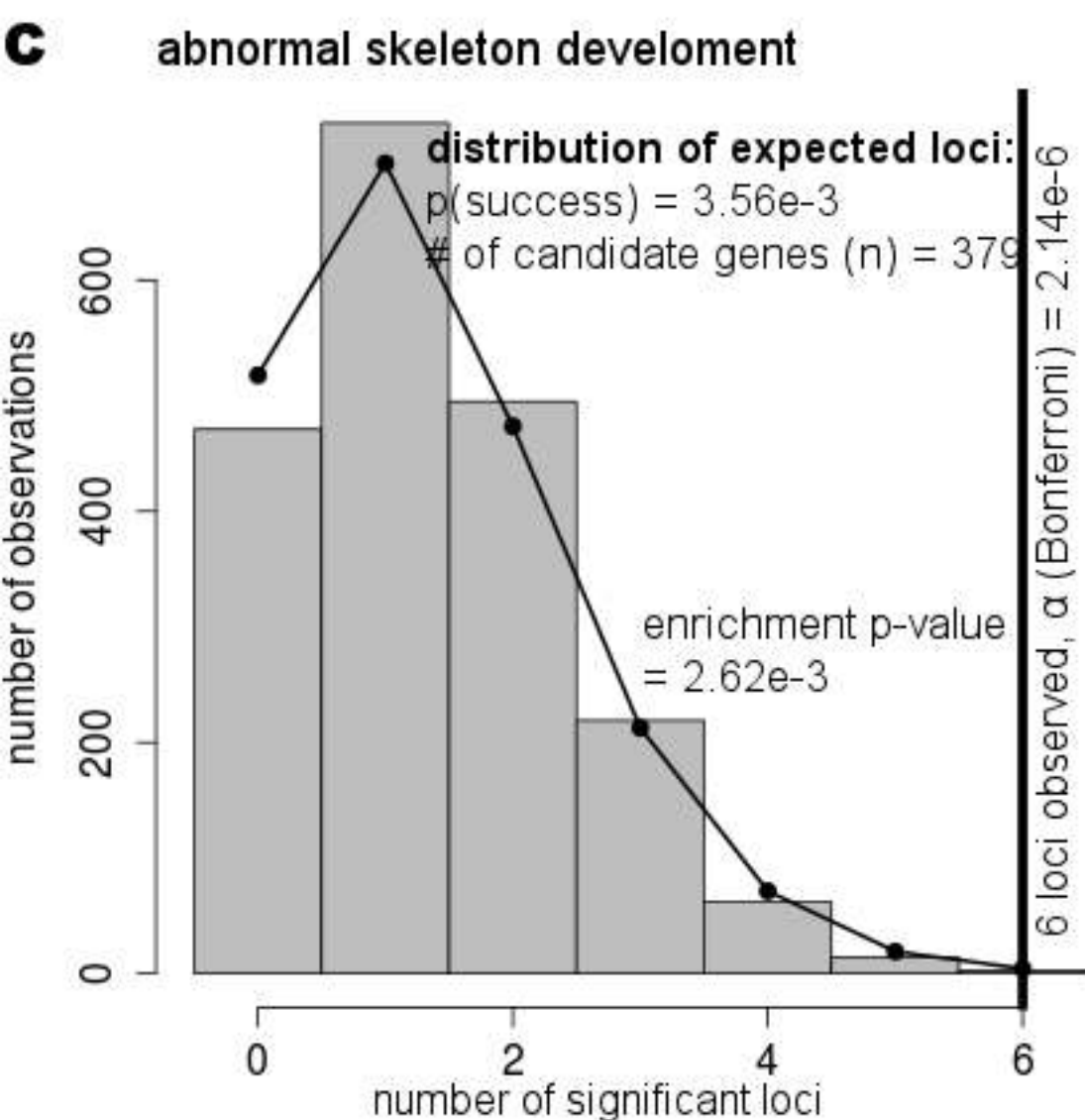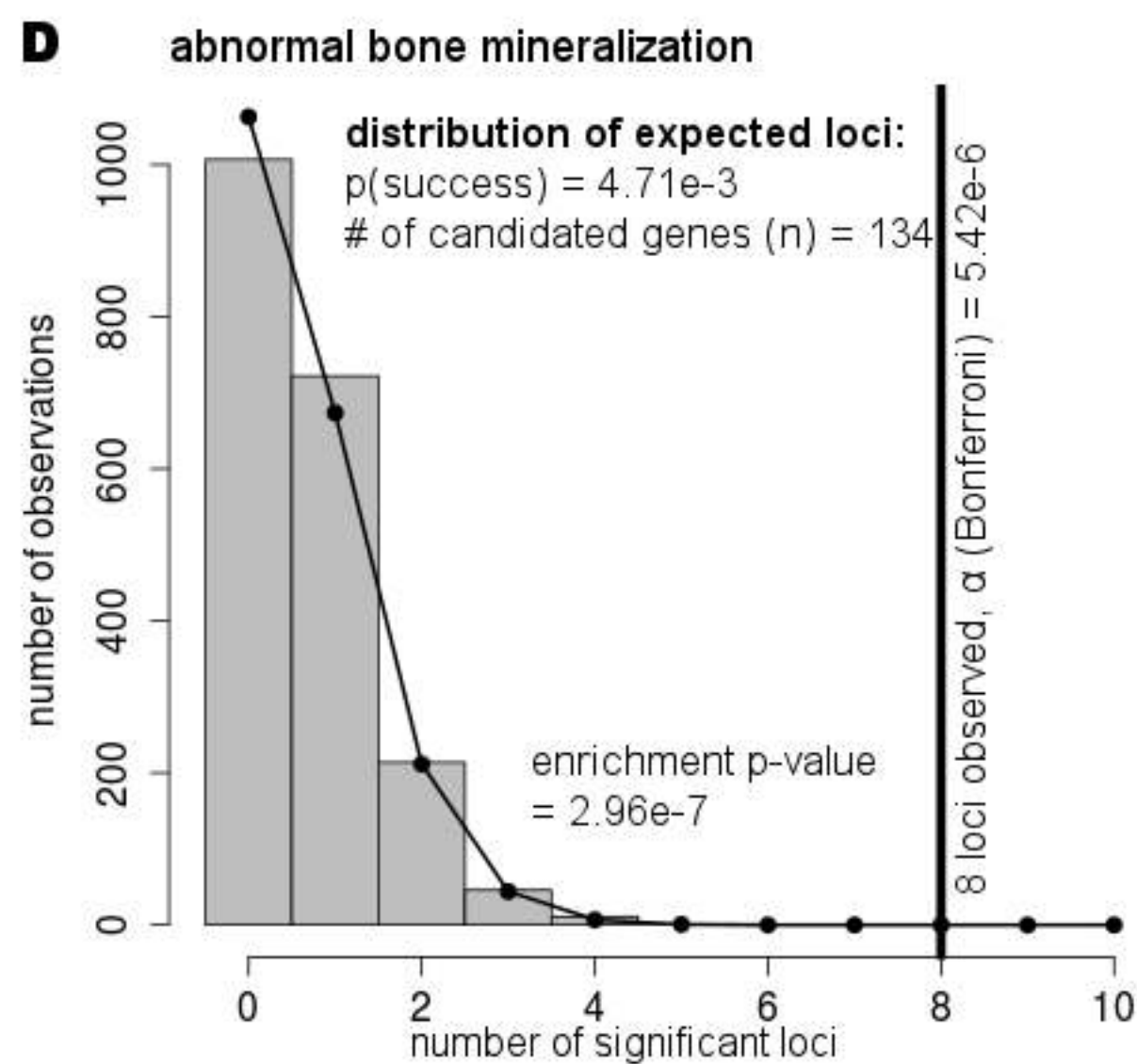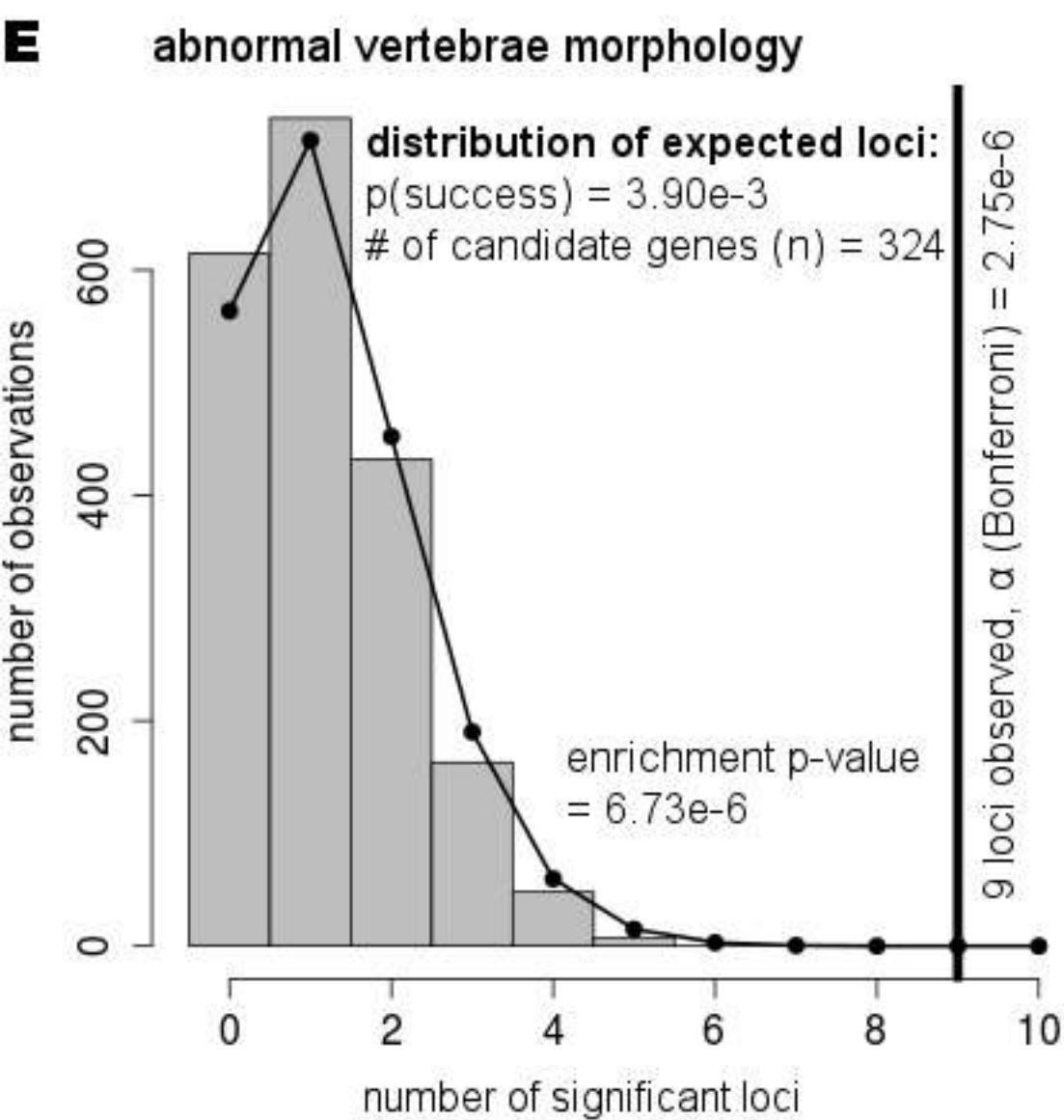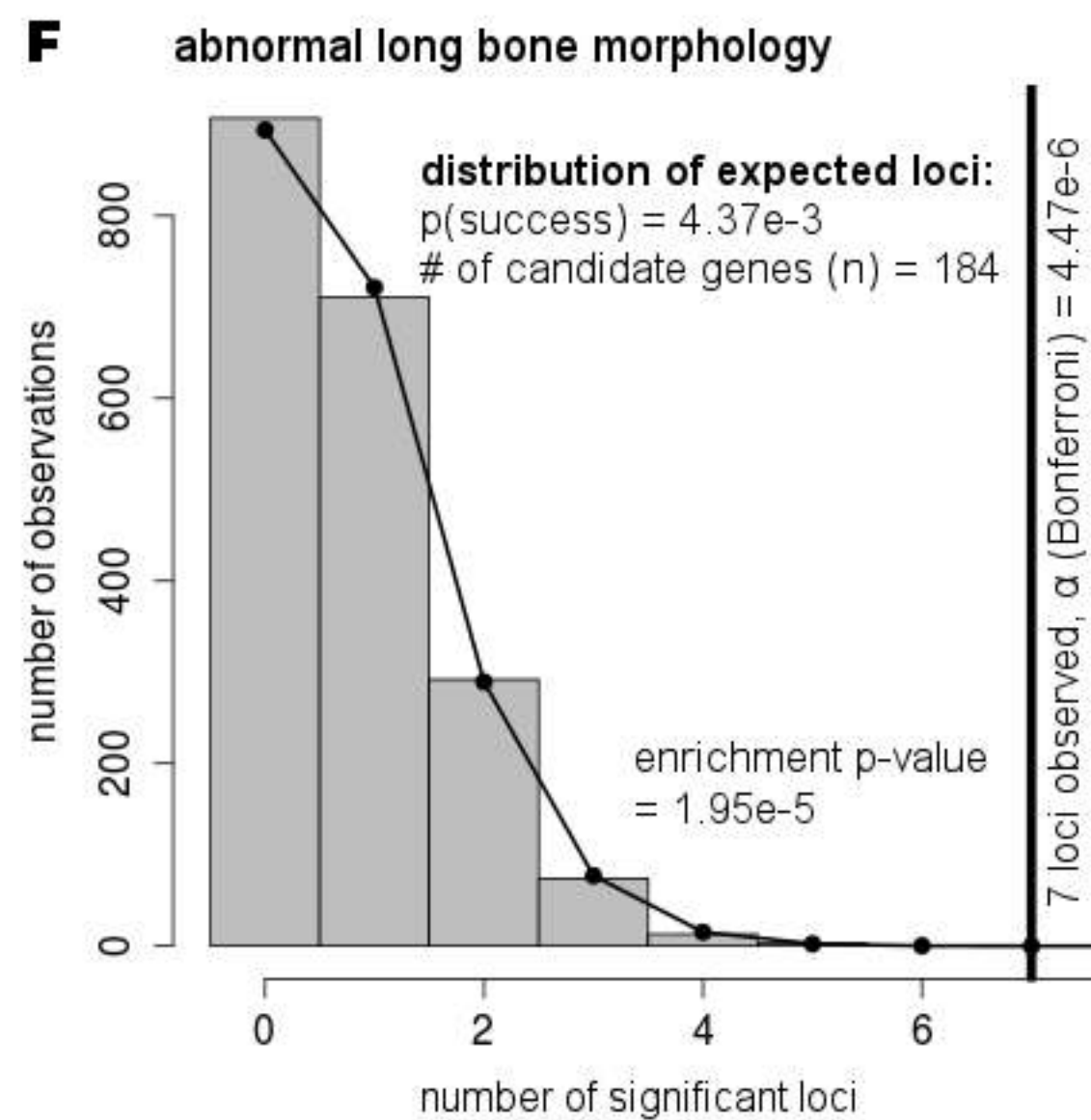

Supplement: S3 Fig — (PDF) [file pone.0162466.s003.pdf]

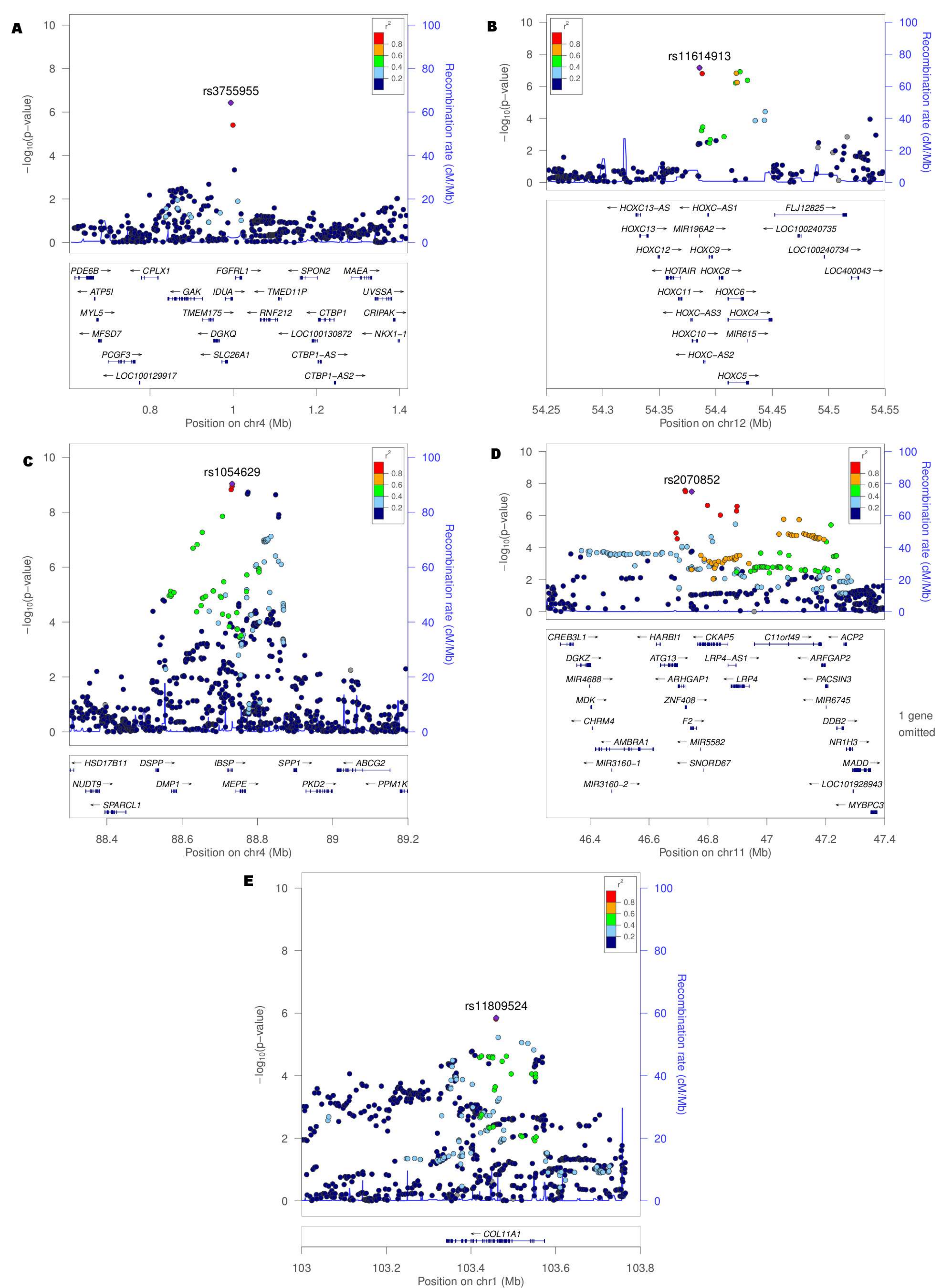

Supplement: S4 Fig — (PDF) [file pone.0162466.s004.pdf]

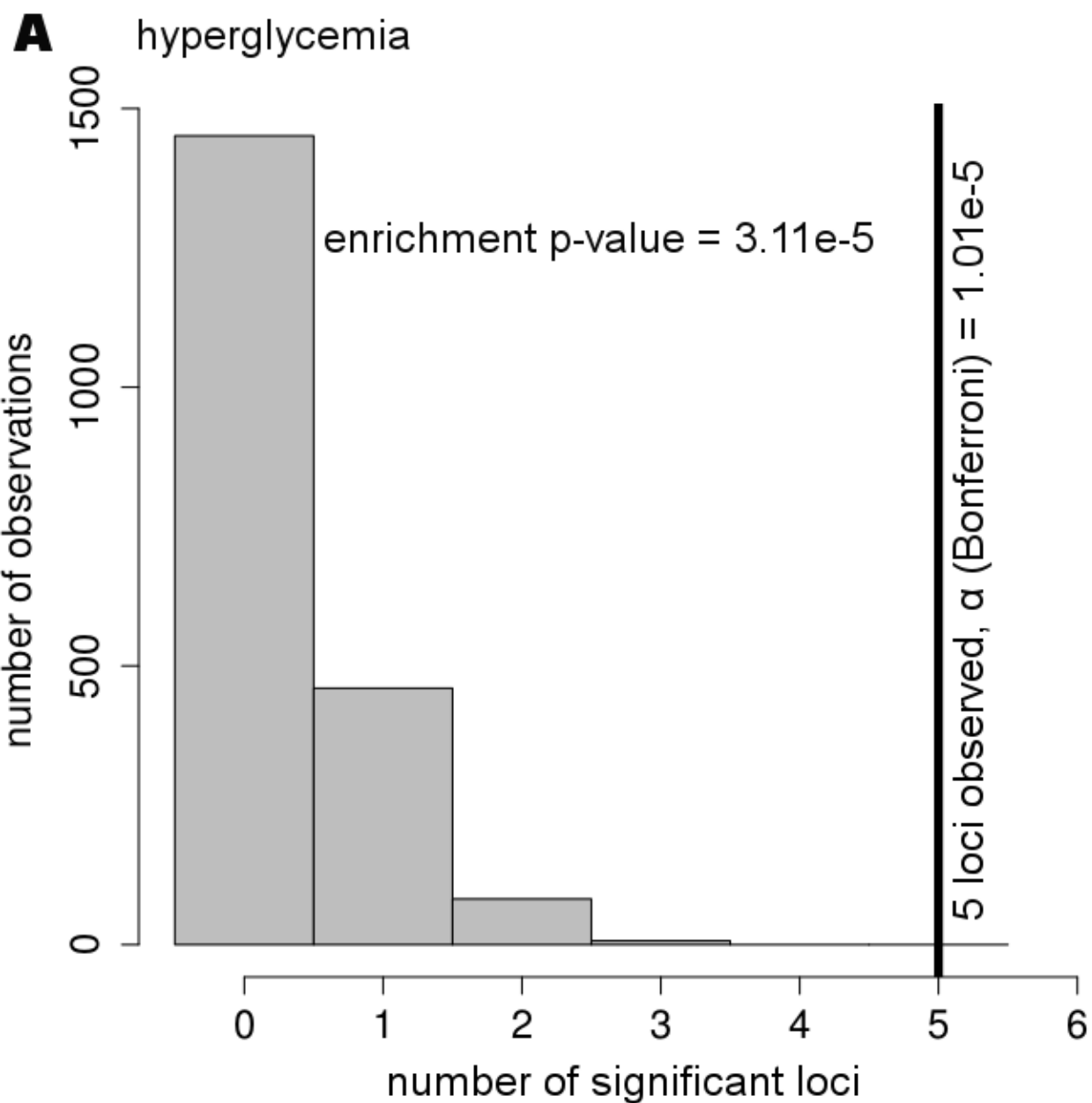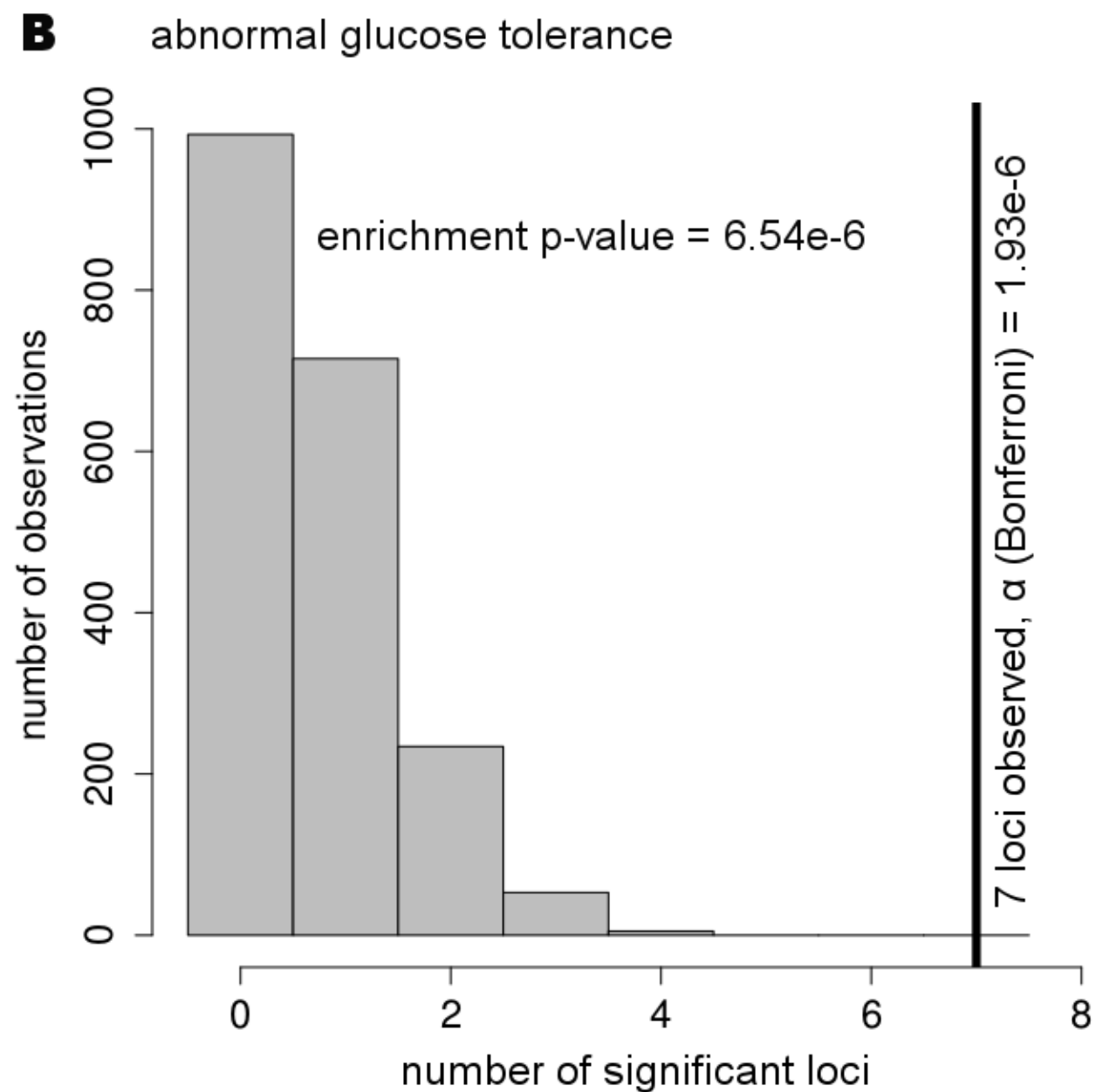

Supplement: S5 Fig — (PDF) [file pone.0162466.s005.pdf]

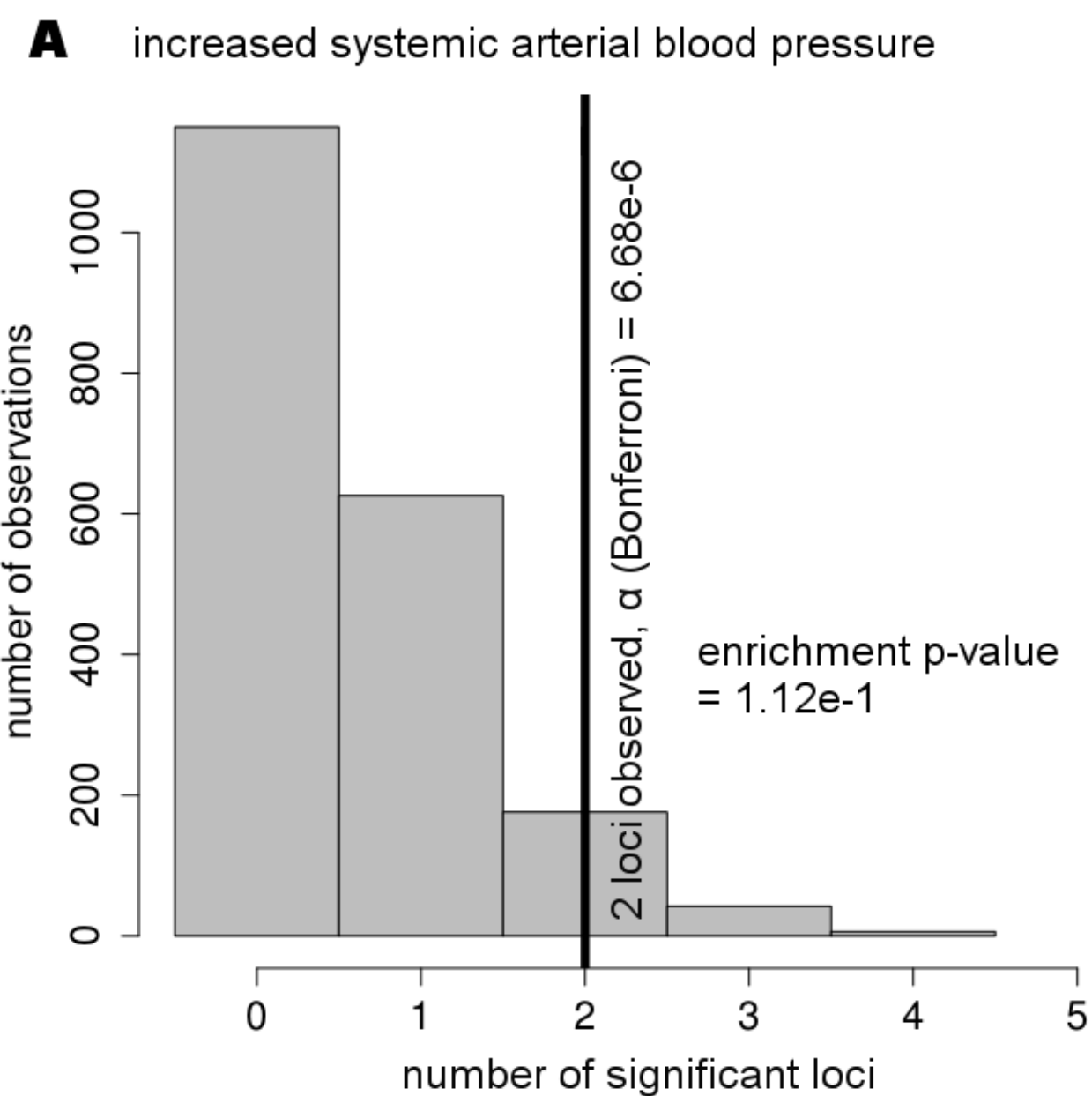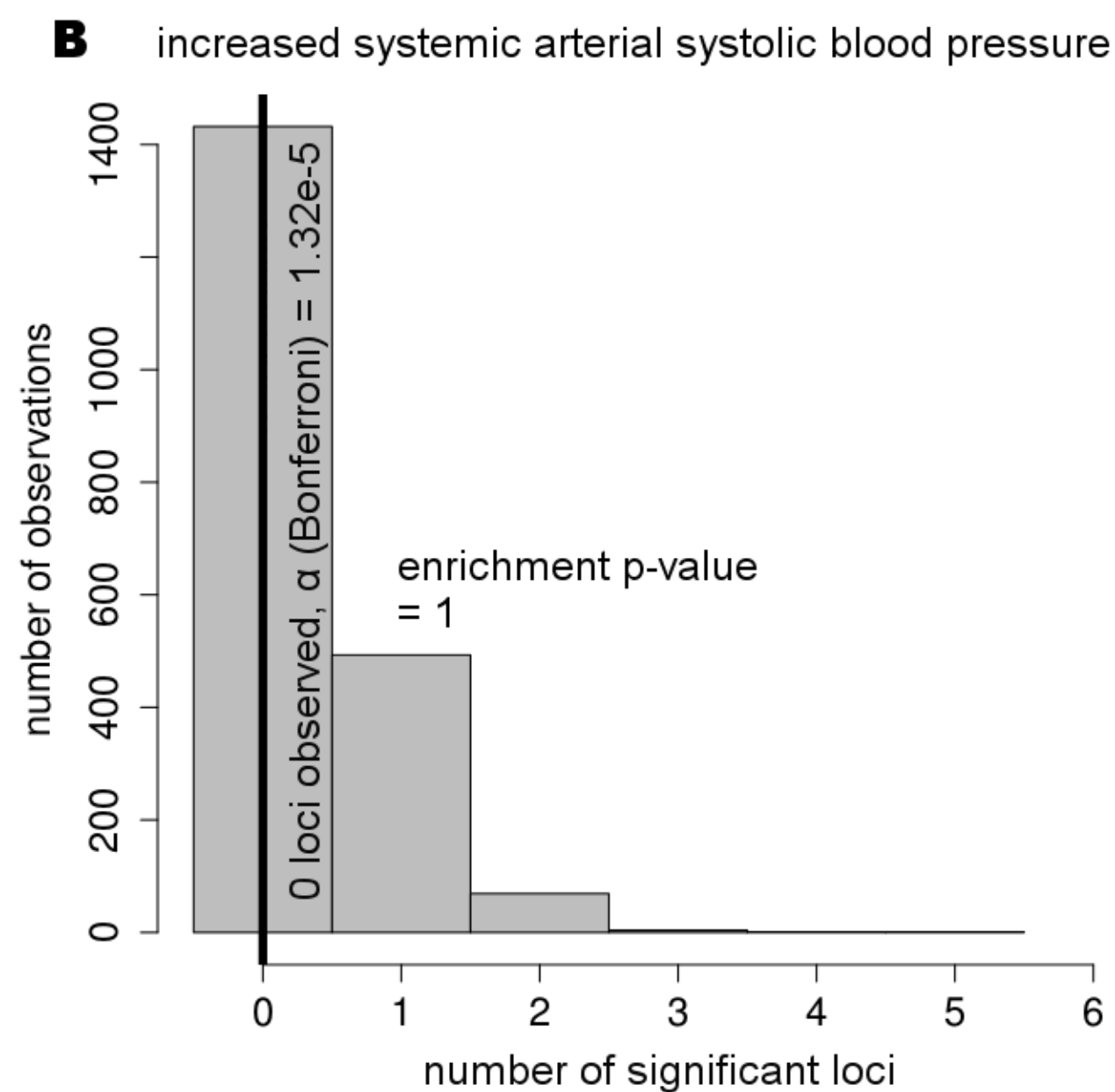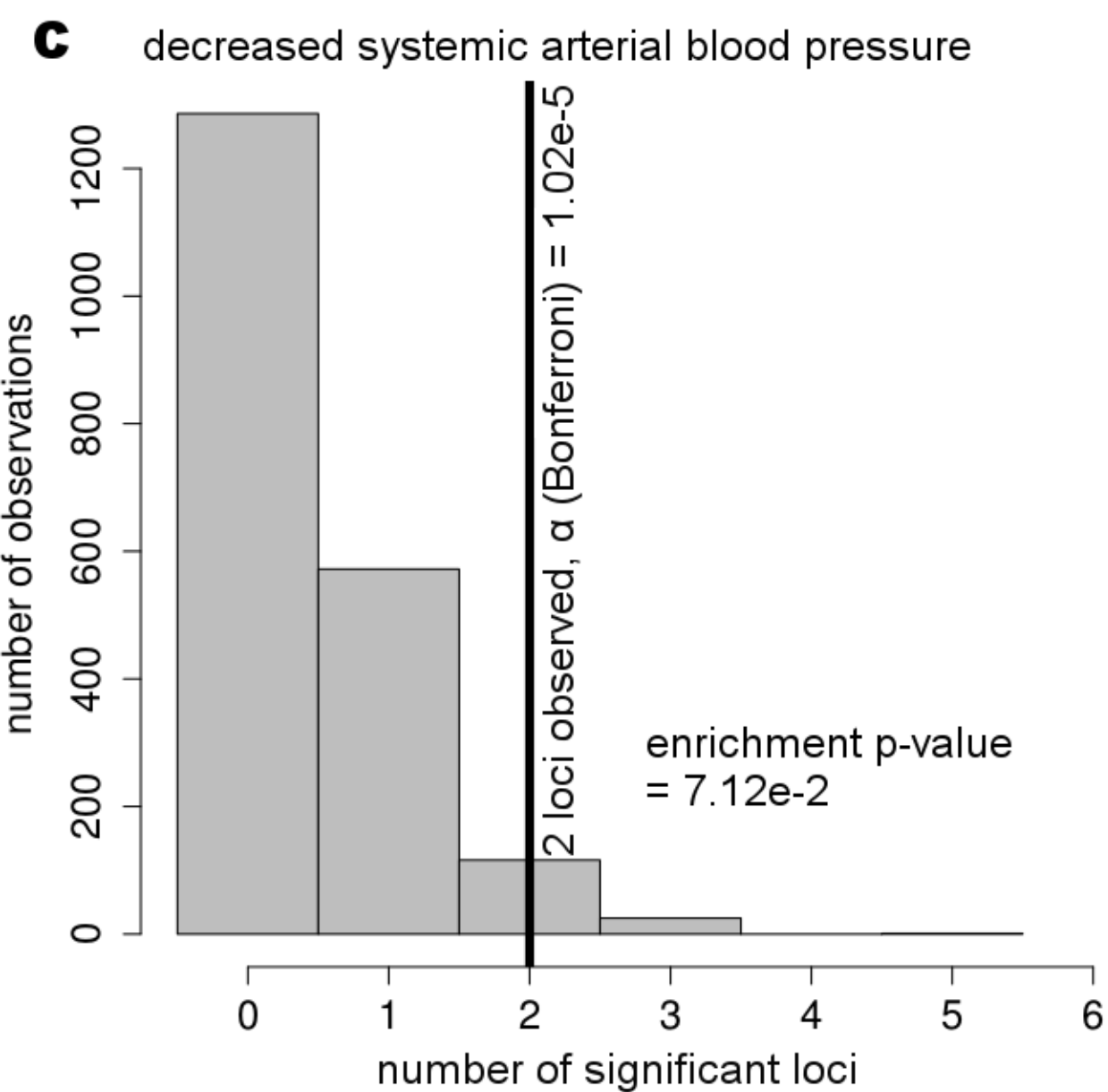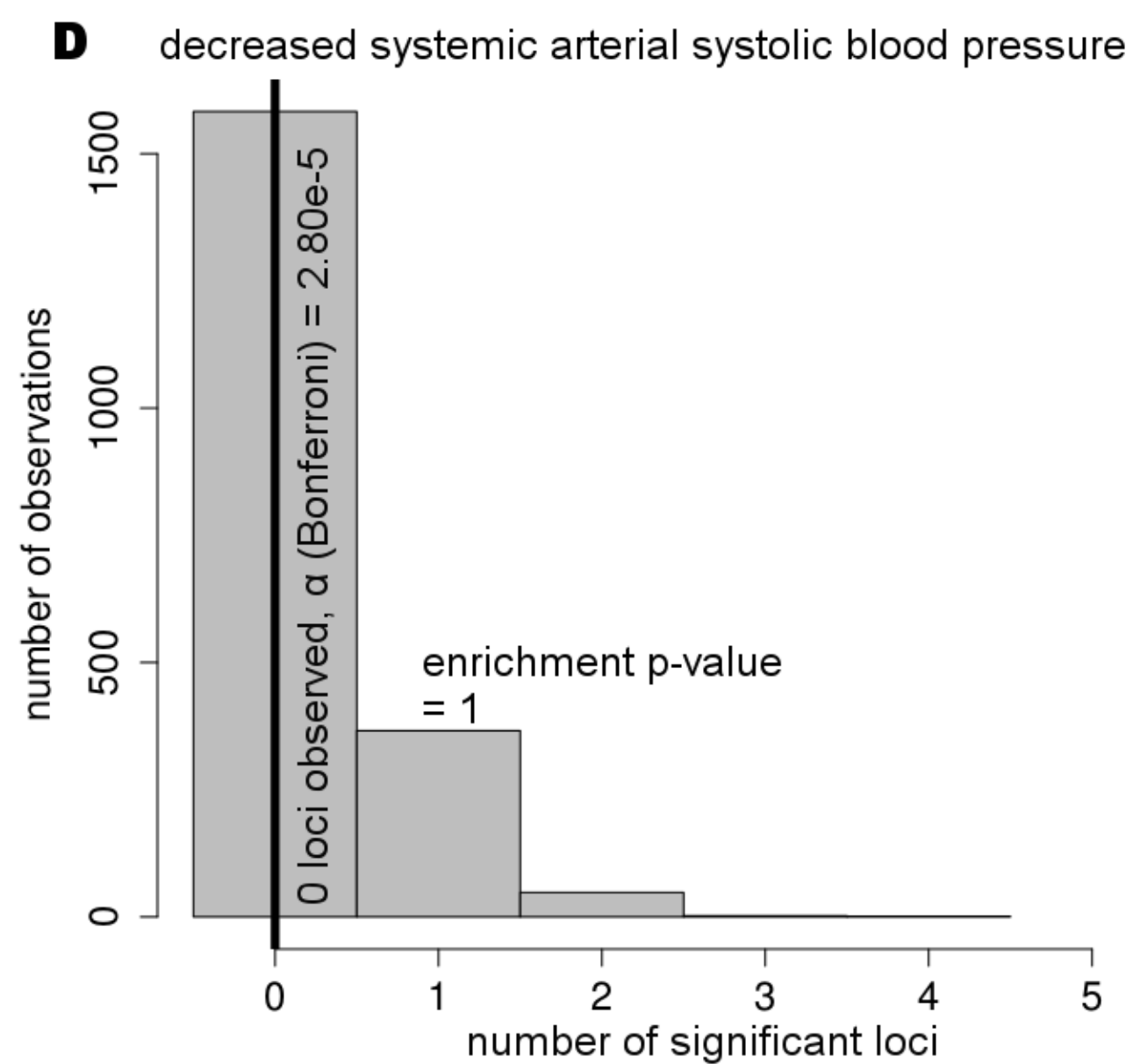

Supplement: S6 Fig — (PDF) [file pone.0162466.s006.pdf]

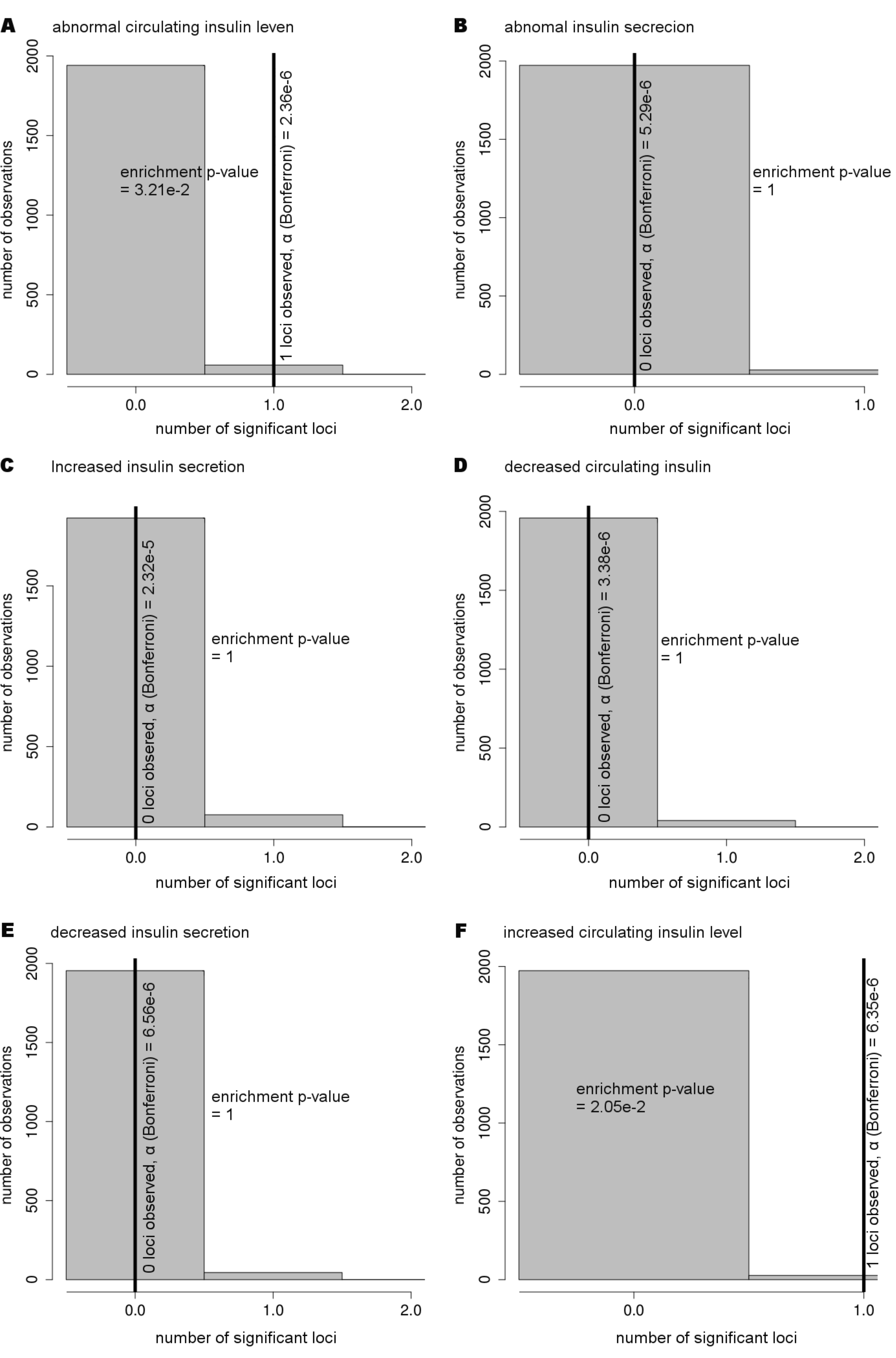

Supplement: S7 Fig — (PDF) [file pone.0162466.s007.pdf]

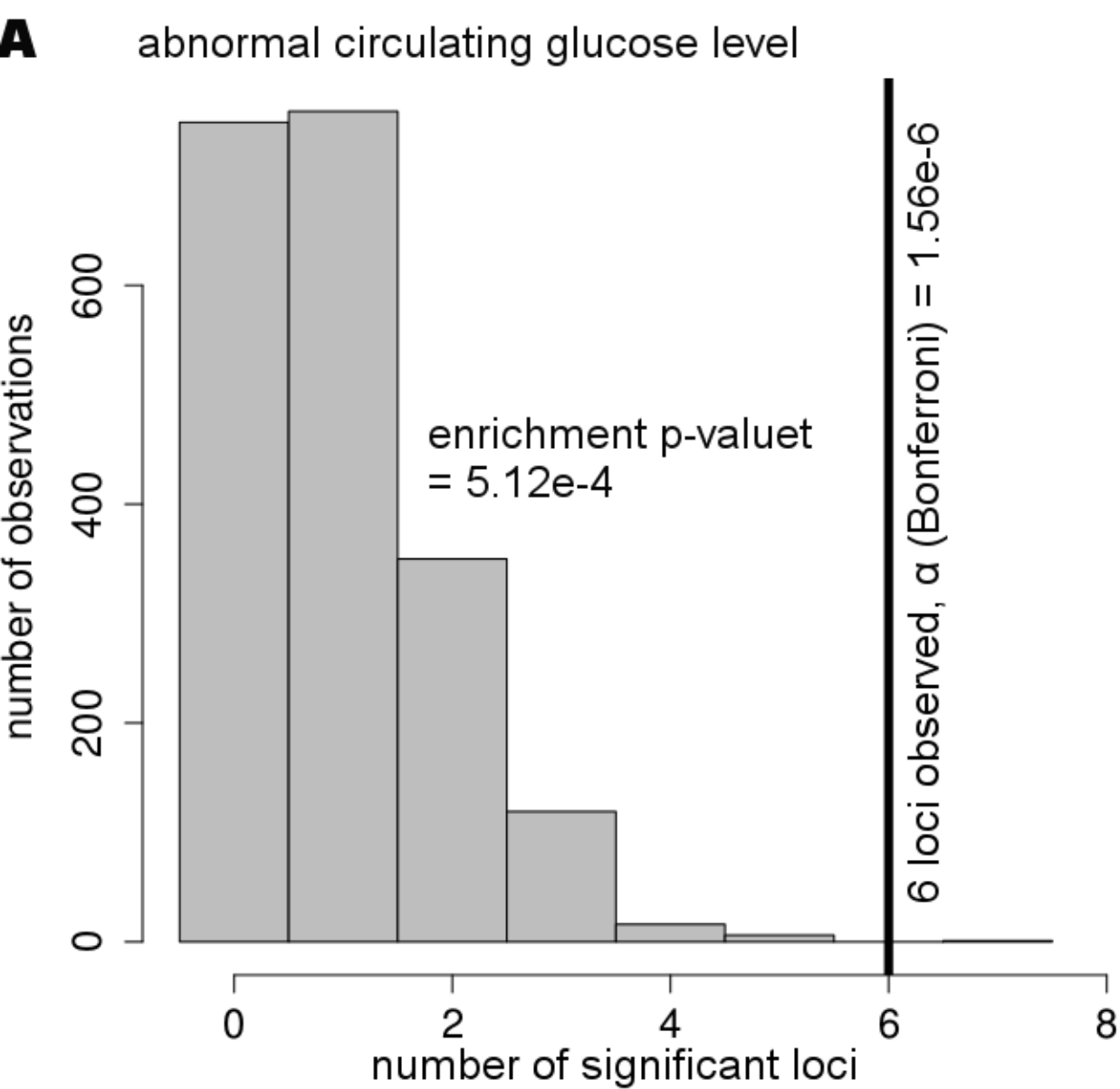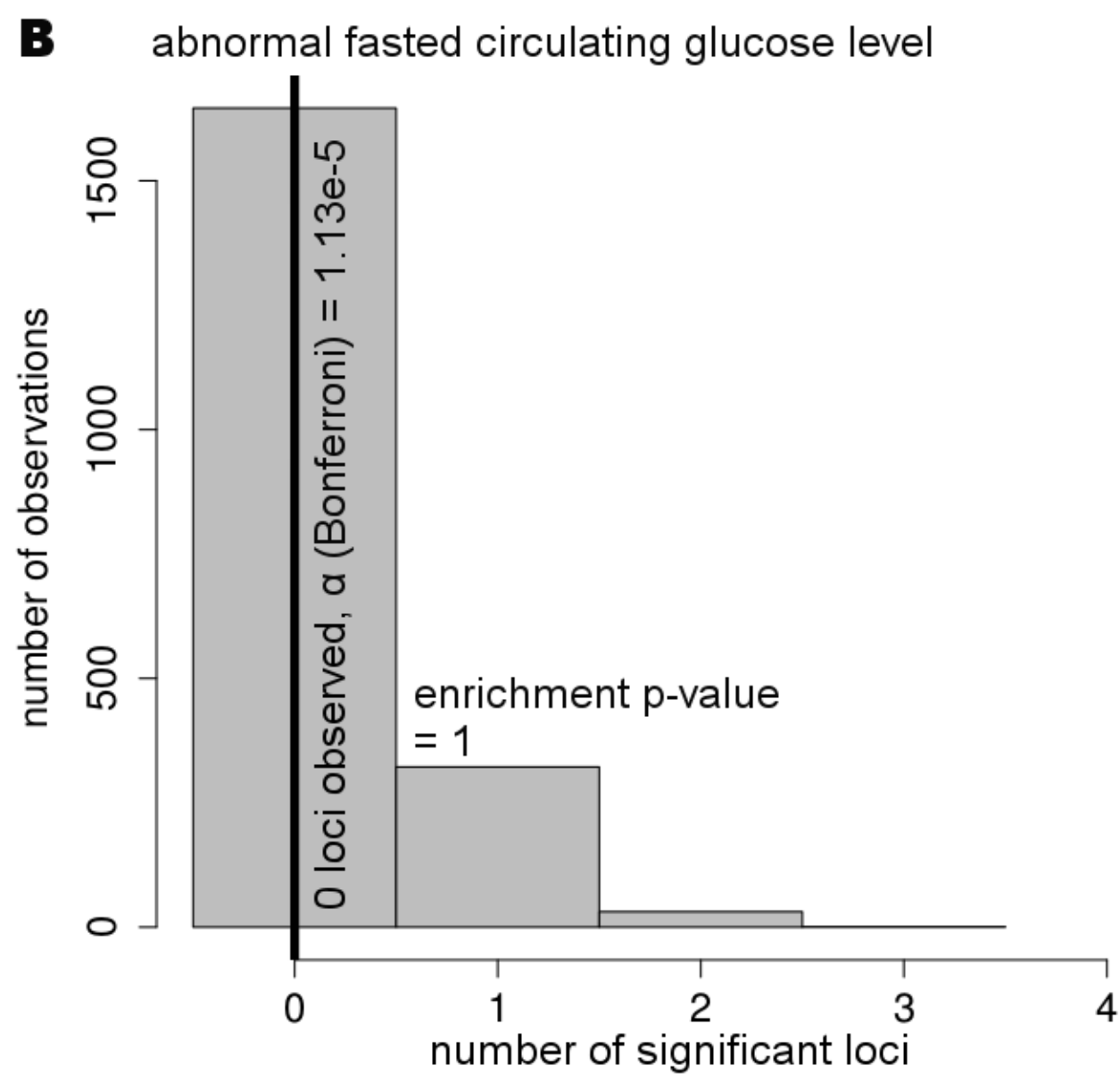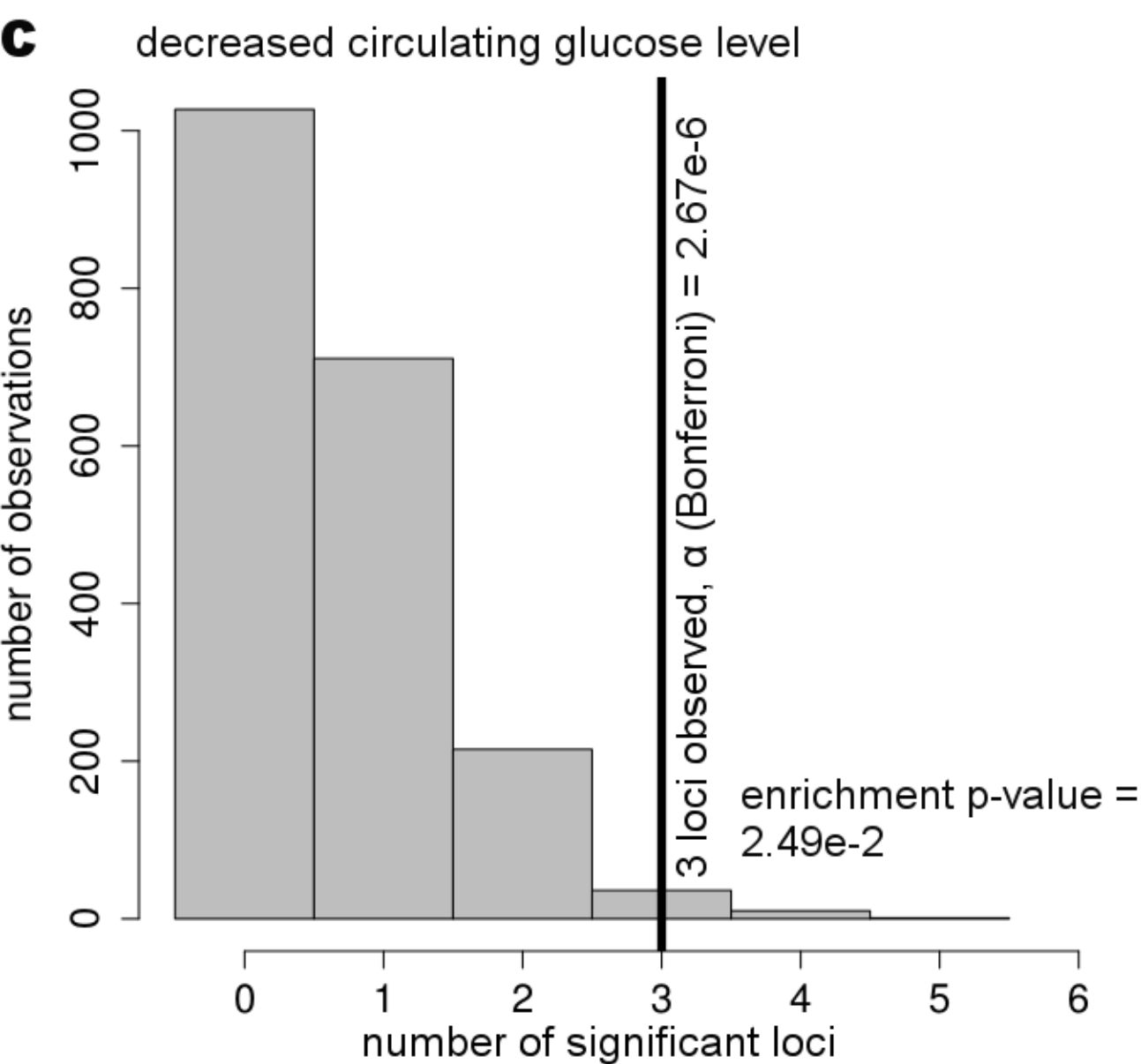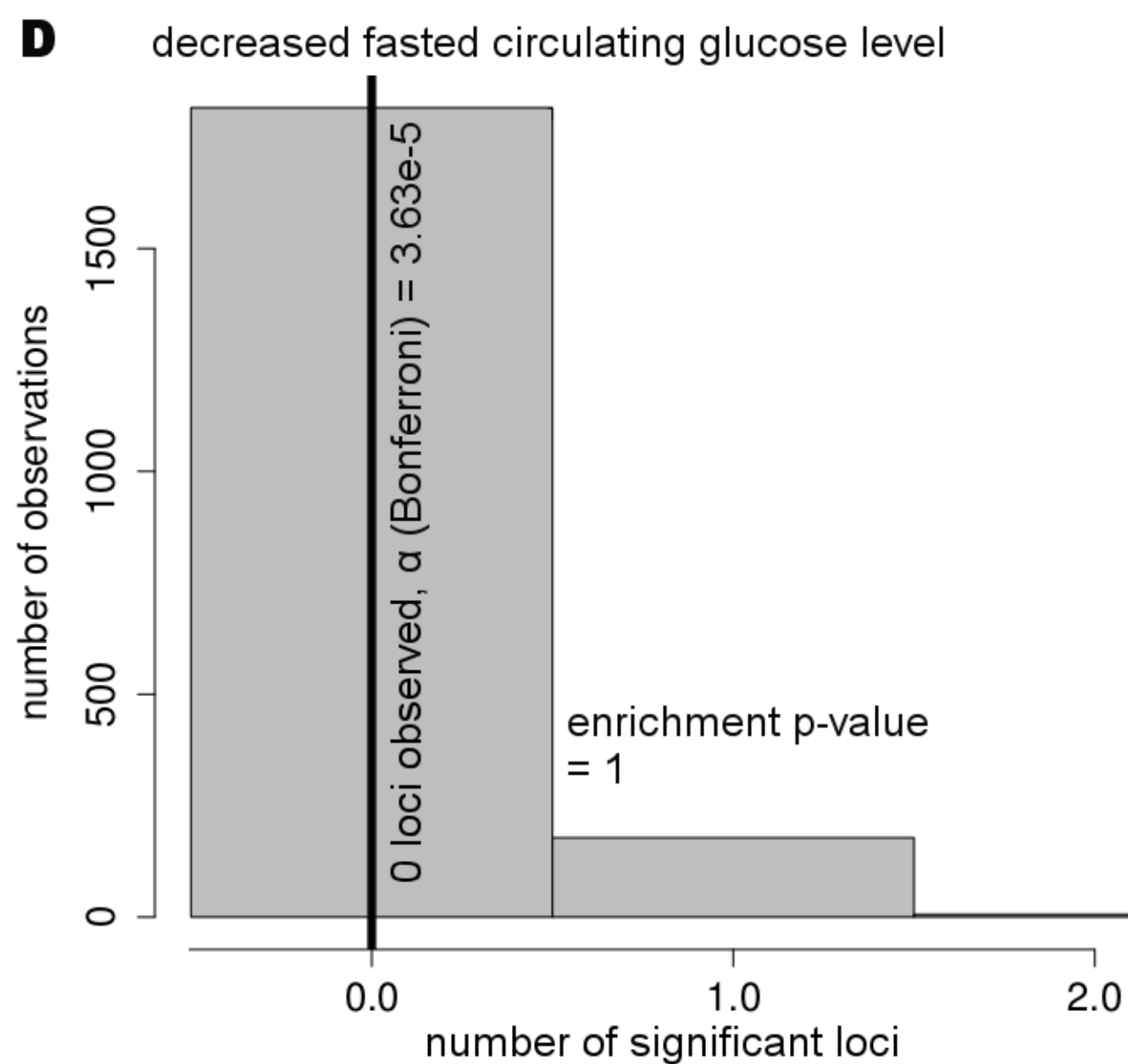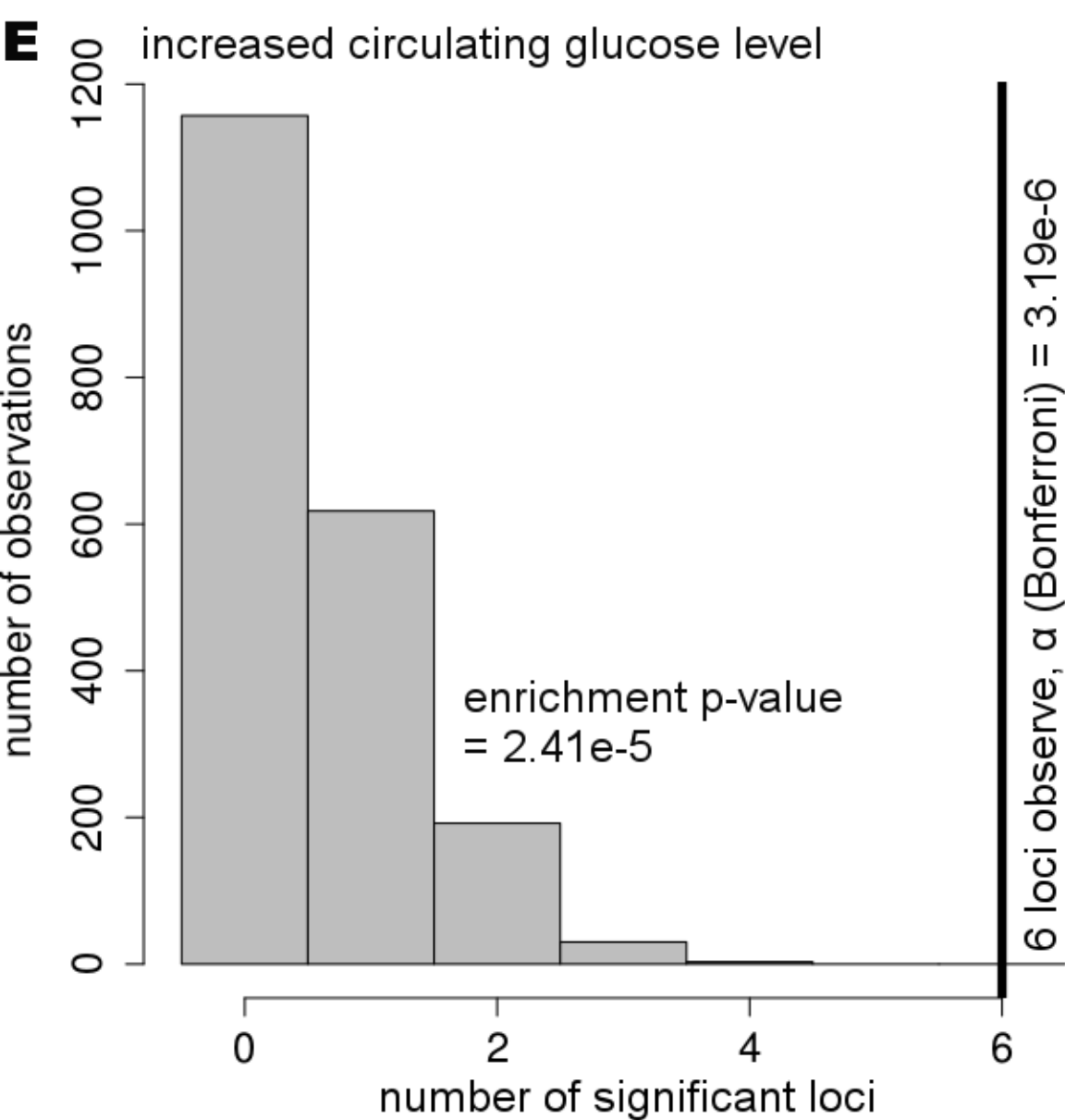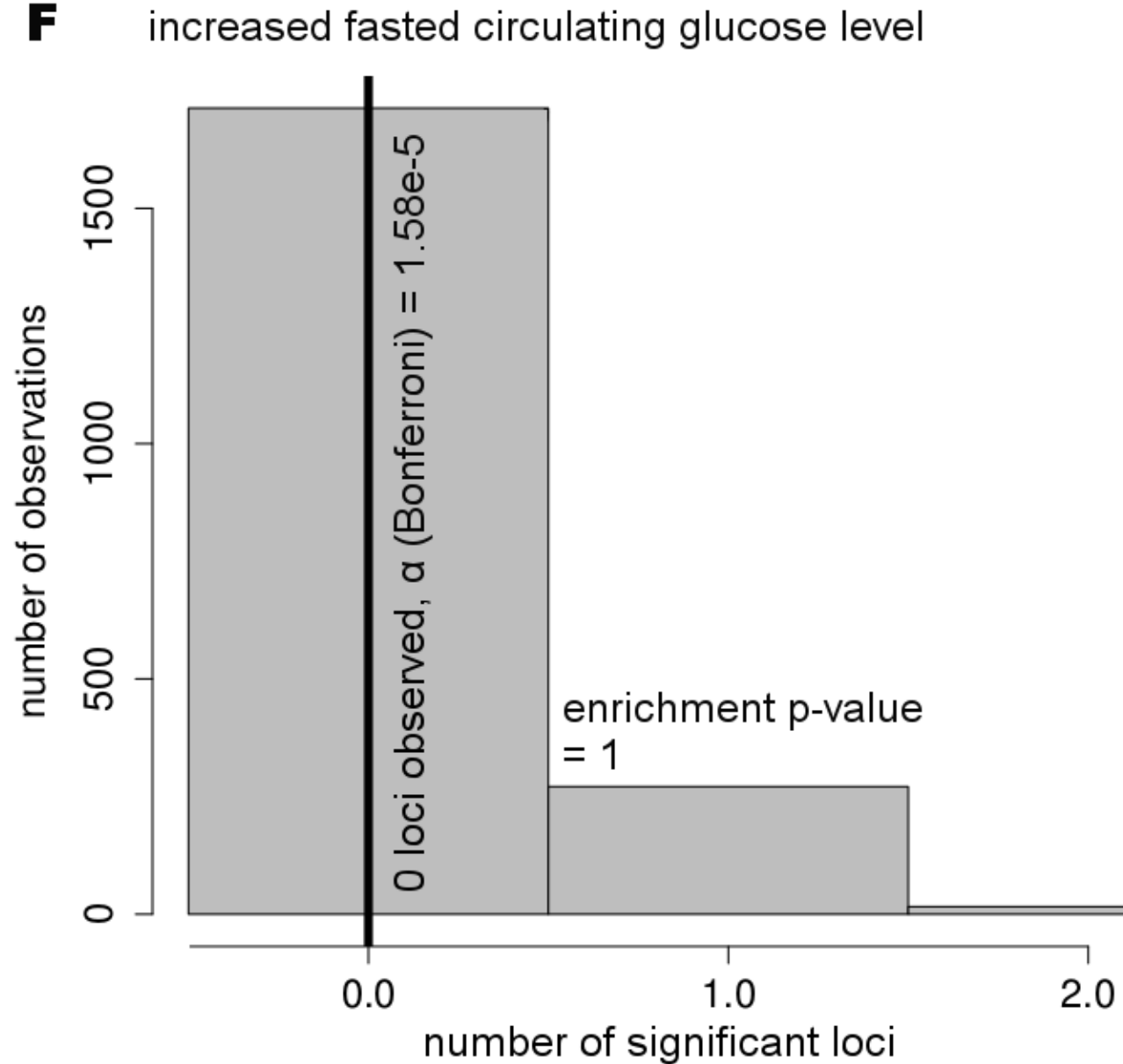

Supplement: S8 Fig — (PDF) [file pone.0162466.s008.pdf]
