# Supplementary material for: GenToS: Use of Orthologous Gene Information to Prioritize Signals from Human GWAS
Source: PLoS One. 2016 Sep 9;11(9):e0162466. doi: 10.1371/journal.pone.0162466 (PMC5017755; doi:10.1371/journal.pone.0162466)
Supplement: S1 Table — (PDF) [file pone.0162466.s009.pdf]

Supplementary Table 1: Genes with significant SNP associations with diabetes, glycemic measures and blood pressure measurements.

DIAGRAM Diabetes					
**Gene**	**index SNP**	**P-value**	**in GWAS Catalog**	**OMIM number**	**monogenic phenotype**
HNF1A	rs12427353	1.0E-6	Other traits	612520<br>142330<br>600496<br>144700<br>222100<br>125853	Diabetes mellitus, insulin-dependent, 20<br>Hepatic adenoma, somatic<br>MODY, type III<br>Renal cell carcinoma<br>{Diabetes mellitus, insulin-dependent}<br>{Diabetes mellitus, noninsulin-dependent, 2}
WFS1	rs1801214	1.3E-8	Type 2 diabetes	222300	Wolfram syndrome
PPARG	rs11709077	1.1E-9	Type 2 diabetes<br>And other traits	609338<br>604367<br>604367<br>601665<br><br>125853	Carotid intimal medial thickness 1<br>Insulin resistance, severe, digenic<br>Lipodystrophy, familial partial, type 3<br>Obesity, severe<br>[Obesity, resistance to]<br>{Diabetes, type 2}
IDE	rs11187007	1.8E-12	Type 2 diabetes<br>And other traits		
KCNJ11	rs5215	4.4E-6	Type 2 diabetes and other traits	606176<br>610582<br>606176<br>601820<br>616329<br>125853	Diabetes mellitus, permanent neonatal, with neurologic features<br>Diabetes mellitus, transient neonatal, 3<br>Diabetes, permanent neonatal<br>Hyperinsulinemic hypoglycemia, familial, 2<br>Maturity-onset diabetes of the young, type 13<br>{Diabetes mellitus, type 2, susceptibility to}
CDKAL1	rs7756992	1.3E-22	Type 2 diabetes<br>Diabetes (gestation)<br>Glycated hemoglobin levels<br>And other traits	125853	{Diabetes mellitus, noninsulin-dependent, susceptibility to}
FTO	rs17817449	5.6E-11	other traits	612938<br>612460	Growth retardation, developmental delay, facial dysmorphism<br>{Obesity, susceptibility to, BMIQ14}
SLC30A8	rs3802177	2.1E-11	Glycolated hemoglobin levels<br>And other traits	125853	{Diabetes mellitus, noninsulin-dependent, susceptibility to}
TCF7L2	rs7901695	2.5E-65	Type 2 diabetes<br>Glycated hemoglobin levels<br>Proinsulin levels<br>Two-hour glucose challenge	125853	{Diabetes mellitus, type 2, susceptibility to}

MAGIC fasting insulin					
**Gene**	**index SNP**	**P-value**	**in GWAS Catalog**	**OMIM number**	**monogenic phenotype**
IGF1	rs35767	9.58E-005	Fasting insulin realted traits<br>Fasting glucose related traits<br>And other traits	608747	Growth retardation with deafness and mental retardation due to IGF1 deficiency
**MAGIC fasting glucose**					
**Gene**	**index SNP**	**P-value**	**in GWAS Catalog**	**OMIM number**	**monogenic phenotype**
G6PC2	rs560887	4.61E-75	Fasting plasma glucose<br>Glycated hemoglobin levels		
GCK	rs1799884	6.36E-37	Fasting plasma glucose<br>Glycated hemoglobin levels	125853<br>606176<br>602485<br>125851	Diabetes mellitus, noninsulin-dependent, late onset<br>Diabetes mellitus, permanent neonatal<br>Hyperinsulinemic hypoglycemia, familial, 3<br>MODY, type II
GCKR	rs780093	2.91E-10	Hyperglyceridemia<br>And other traits	613463	[Fasting plasma glucose level QTL 5]
SLC2A2	rs11920090	1.87E-6	Fasting glucose realted traits<br>And other traits	227810<br>125853	Fanconi-Bickel syndrome<br>{Diabetes mellitus, noninsulin-dependent}
SLC30A8	rs11558471	2.62E-8	Glycolated hemoglobin levels<br>And other traits	125853	{Diabetes mellitus, noninsulin-dependent, susceptibility to}
TCF7L2	rs4506565	1.24E-5	Type 2 diabetes<br>Glycated hemoglobin levels<br>Proinsulin levels<br>Two-hour glucose challenge	125853	{Diabetes mellitus, type 2, susceptibility to}
WFS1	rs10012946	4.17E-7	Type 2 diabetes	222300	Wolfram syndrome
**ICBP systolic blood pressure**					
**Gene**	**index SNP**	**P-value**	**in GWAS Catalog**	**OMIM number**	**monogenic phenotype**
ADM	rs1450266	1.56E-6	Diastolic blood pressure<br>Systolic blood pressure		
NPPA	rs5068	1.86E-7	Systolic blood pressure	612201<br>615745	Atrial fibrillation, familial, 6<br>Atrial standstill 2
NPR3	rs1173747	7.38E-7	Blood pressure<br>And other traits		?Hypertension, salt-resistant
PTPN11	rs11066320	4.56E-8	Blood pressure<br>Hematological parameters<br>And other traits	151100<br>607785<br>156250<br>163950	LEOPARD syndrome 1<br>Leukemia, juvenile myelomonocytic, somatic<br>Metachondromatosis<br>Noonan syndrome 1
